# Tyrosine Phosphorylation Allows Integration of Multiple Signaling Inputs by IKKβ

**DOI:** 10.1371/journal.pone.0084497

**Published:** 2013-12-27

**Authors:** April N. Meyer, Kristine A. Drafahl, Christopher W. McAndrew, Jennifer E. Gilda, Leandro H. Gallo, Martin Haas, Laurence M. Brill, Daniel J. Donoghue

**Affiliations:** 1 Department of Chemistry and Biochemistry, University of California San Diego, La Jolla, California, United States of America; 2 Moores UCSD Cancer Center, University of California San Diego, La Jolla, California, United States of America; 3 Sanford-Burnham Medical Research Institute, La Jolla, California, United States of America; University of Virginia, United States of America

## Abstract

Signaling regulated by NFκB and related transcription factors is centrally important to many inflammatory and autoimmune diseases, cancer, and stress responses. The kinase that directly regulates the canonical NFκB transcriptional pathway, Inhibitor of κB kinase β (IKKβ), undergoes activation by Ser phosphorylation mediated by NIK or TAK1 in response to inflammatory signals. Using titanium dioxide-based phosphopeptide enrichment (TiO_2_)-liquid chromatography (LC)-high mass accuracy tandem mass spectrometry (MS/MS), we analyzed IKKβ phosphorylation in human HEK293 cells expressing IKKβ and FGFR2, a Receptor tyrosine kinase (RTK) essential for embryonic differentiation and dysregulated in several cancers. We attained unusually high coverage of IKKβ, identifying an abundant site of Tyr phosphorylation at Tyr169 within the Activation Loop. The phosphomimic at this site confers a level of kinase activation and NFκB nuclear localization exceeding the iconic mutant S177E/S181E, demonstrating that RTK-mediated Tyr phosphorylation of IKKβ has the potential to directly regulate NFκB transcriptional activation.

## Introduction

Receptor tyrosine kinases (RTKs) represent important signal transducers in the cell membrane and are comprised of nearly twenty families of homologous proteins in humans, with almost 60 distinct members [1]. In the FGFR family, four homologous human receptors have been identified: FGFR1, FGFR2, FGFR3 and FGFR4. All of the FGFRs exhibit three extracellular immunoglobulin (Ig)-like domains, a membrane-spanning segment and a split tyrosine kinase domain [2]. In embryonic development, FGFRs play crucial roles in mitogenesis, migration, and cell proliferation, while in adult organisms, FGFRs are majorly responsible for tissue repair and wound healing [3]. Specific mutations in the human genes encoding FGFR1, FGFR2, or FGFR3 lead to congenital bone diseases classified as chondrodysplasia and craniosynostosis syndromes, which cause dwarfism, deafness, and abnormalities of the skeleton, skin and eye. FGFR2 signaling has also been found to be important in many human cancers, such as prostate cancer, bladder cancer, gastric cancer, breast cancer and melanoma [4-8]. 

NFκB signaling, of central importance in human disease [9-11], is regulated by a complex composed of Inhibitor of κB Kinase α (IKKα), IKKβ and the scaffolding protein IKKγ/NEMO [12-15]. Constitutive activation of IKKβ has been implicated in many diseases, including multiple myeloma, breast and ovarian cancers, as well as rheumatoid arthritis, insulin resistance, and liver degeneration [16-19]. Multiple myeloma (MM) is a severe and incurable malignancy of B-lymphoid cells in which malignant progression has been linked to the activation of various pathways, including NF-κB [10,18]. The upregulation of IKKβ has also been implicated in rheumatoid arthritis (RA) [20]. More specifically, IKKβ has been show to regulate IL1- and TNFα-induced expression of ICAM-1 and collagenase synthesis in RA synoviocytes [21]. The primary regulatory kinase of the canonical NFκB transcriptional pathway, IKKβ, undergoes activation by Ser phosphorylation within the Activation Loop mediated by NIK or TAK1 in response to inflammatory signals such as TNFα, IL-1 or LPS [22-25]. Once activated, IKKβ phosphorylates IκBα at S32/S36, which is subsequently polyubiquitinated and degraded by the 26S-proteasome [26]. Once IκBα is degraded, the nuclear localization signal of NFκB (RelA:p50) triggers the nuclear translocation of this transcription factor, which binds to the κB promoter of genes involved in inflammation, immunity, cell growth, differentiation and survival [27,28].

We undertook an analysis of RTK-stimulated phosphorylation of IKKβ using titanium dioxide-based phosphopeptide enrichment (TiO_2_)-liquid chromatography (LC)-high mass accuracy tandem mass spectrometry (MS/MS) [29,30], attaining unusually robust coverage of IKKβ. In particular, the most abundant site of Tyr phosphorylation, Tyr169 within the Activation Loop, when mutated to its phosphomimic confers a level of kinase activation and NFκB nuclear localization exceeding the iconic S177E/S181E “EE” mutant [24]. 

## Results

**Figure 1:**
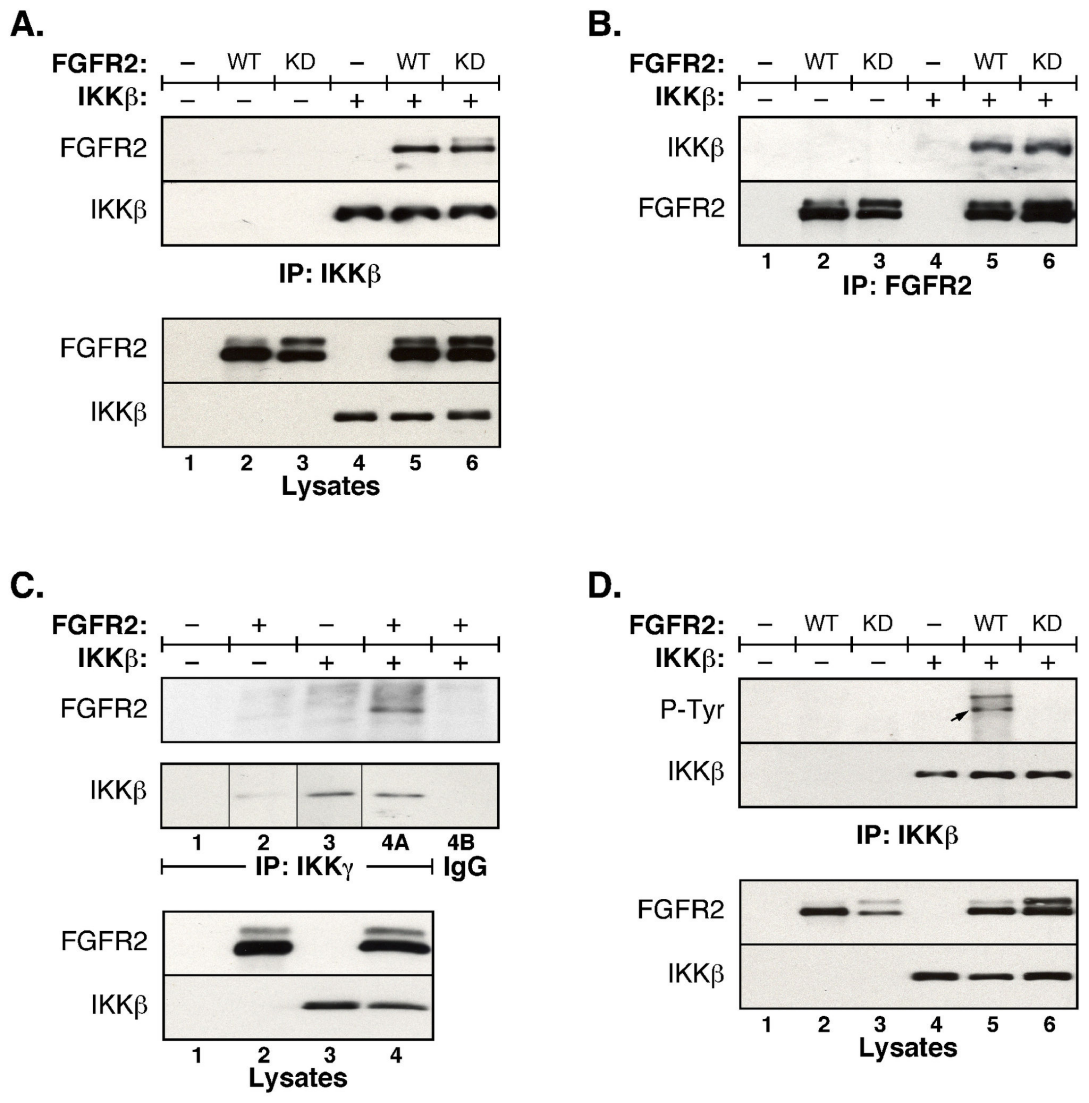
FGFR2 interacts with IKKβ and stimulates tyrosine phosphorylation of IKKβ. FGFR2 wildtype (WT) or kinase dead (KD) and IKKβ were expressed in HEK293 cells. (**A**) **FGFR2 associates with IKKβ**. IKKβ was immunoprecipitated from lysates and analysed for FGFR2 by immunoblot (top panel). The membrane was stripped and reprobed for IKKβ (second panel). Expression of FGFR2 and IKKβ is shown in cell lysates (lower panels). (**B**) **IKKβ associates with FGFR2**. FGFR2 was immunoprecipitated from lysates and analysed for IKKβ by immunoblot (top panel). The membrane was stripped and reprobed for FGFR2 (second panel). Lysate blots are as in (A). (**C**) **FGFR2 and IKKβ are present in complexes with IKKγ/NEMO**. Endogenous IKKγ/NEMO was immunoprecipitated from cell lysates expressing FGFR2 and IKKβ using IKKγ/NEMO antisera. The interaction with FGFR2 (top panel) and IKKβ (second panel) was detected by immunoblot. Negative IgG control shown in lane 4B. Note that the 1^st^ and 2^nd^ panels represent duplicate gels of the same samples. The thin black lines on the 2^nd^ panel indicate where additional IgG controls were run but removed from the final figure except for Lane 4B. All samples on this panel are from the same exposure of the same immunoblot. Expression of FGFR2 and IKKβ shown in total lysate (lower panels). (**D**) **FGFR2 stimulates tyrosine phosphorylation of IKKβ**. IKKβ was immunoprecipitated from lysates and analysed by phophotyrosine immunoblot (top panel). The membrane was stripped and reprobed for IKKβ (second panel). Expression of FGFR2 derivatives and IKKβ is shown in total lysates (lower panels). The arrow in Lane 5 of the upper panel indicates IKKβ.

Previously, we identified FGFR4 as a two-hybrid binding partner of IKKβ and showed that FGFR4 modulates TNFα-stimulated NFκB signaling [31]. Here, we show that FGFR2 similarly interacts with IKKβ, as shown by coimmunoprecipitations in which FGFR2 can be detected in IKKβ immune complexes (Figure 1A), or IKKβ can be detected in FGFR2 immune complexes (Figure 1B). Interestingly, the association of FGFR2 with IKKβ is observed even when using the K517R kinase-dead mutant FGFR2 (Lane 6), showing that the interaction can occur in the absence of phosphorylation by FGFR2. Both proteins can also be detected in immune complexes of IKKγ/NEMO (Figure 1C). As we previously demonstrated for FGFR4 [31], FGFR2 also results in Tyr phosphorylation of IKKβ (Figure 1D, Lane 5), which did not occur in response to the kinase-dead FGFR2 (Figure 1D, Lane 6).

The experiments in Figure 1 and throughout this work were conducted using non-epitope-tagged derivatives of IKKβ. Many prior studies of IKKβ have used N-terminal hemagglutinin (HA)-tagged derivatives [24,32,33]. Initially, we were concerned that the high levels of Tyr phosphorylation observed in the presence of FGFRs might be due to the adventitious phosphorylation of multiple Tyr residues within the HA-tag (YPYDVPDYA) [34]. Removal of the HA-tag, and subsequent identification of *bona fide* phosphorylation sites by mass spectrometry was required to authenticate our original observations [31].

The two-hybrid interacting domain of IKKβ (aa 560-756) that was originally identified [31] contains a Helix-Loop-Helix (HLH) domain (aa 605-644) within the Scaffold and Dimerization Domain (SDD) as well as the NEMO Binding Domain (NBD) (aa 705-742) [35]. In binding experiments shown in Figure S1 the HLH domain, tagged with Maltose Binding Protein (MBP), was assayed for association with different GST (Gutathione-S-Transferase)-tagged domains of FGFR2. These experiments identified the distal portion of the FGFR2 kinase domain (KD2, aa 598-757) as binding directly to IKKβ.

**Figure 2:**
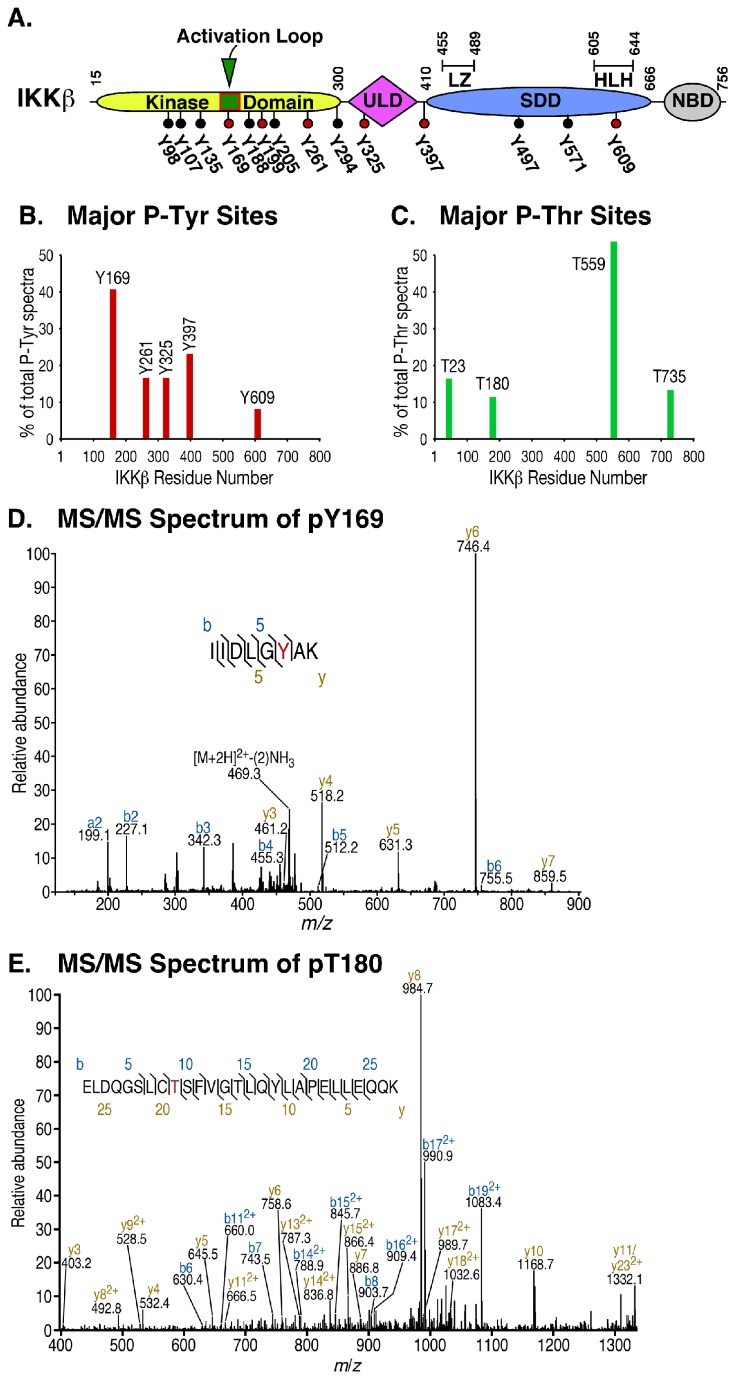
Mass Spectrometry analysis identifies novel phospho-acceptor sites on IKKβ. (**A**) **IKKβ schematic showing tyrosine residues**. A schematic of IKKβ is shown with the N-terminal kinase domain, the ubiquitin-like domain (ULD), the scaffold/dimerization domain (SDD) which also contains the leucine zipper (LZ) and helix-loop-helix (HLH) regions, and the NEMO binding domain (NBD) at the C-terminus of IKKβ [35]. The location of all IKKβ tyrosine residues is shown. Tyrosine residues identified as phosphorylated by TiO_2_-LC-MS/MS analysis are indicated in Red (see Table 1). (**B**) **Major P-Tyr sites identified by MS/MS**. HEK293 cells expressing FGFR2 and IKKβ were collected in RIPA Lysis Buffer, and IKKβ immunoprecipitates were collected, treated with trypsin or pepsin, and resulting peptides prepared for MS/MS analysis as described in Materials and Methods. Spectral counts of major phospho-Tyr residues detected are presented in this graph as a percentage of the total number of phospho-Tyr peptide spectra detected for IKKβ in this study (See Table 1). (**C**) **Major P-Thr sites identified by MS/MS**. Spectral counts of major phospho-Thr residues detected are presented in this graph as a percentage of the total number of phospho-Thr peptide spectra detected for IKKβ in this study (see Table S2). (**D**) **Analysis of pY169**. A definitive MS/MS spectrum of the peptide containing phospho-Tyr169 is presented. (**E**) **Analysis of pT180**. A definitive MS/MS spectrum of the peptide containing phospho-Thr180 is presented.

The primary focus of this work was to identify sites of tyrosine phosphorylation within IKKβ with biological significance. The location of all 14 individual Tyr residues is shown with regard to major domains of IKKβ (Figure 2A). Table S1 summarizes data from six different experiments in which FGFR2 and IKKβ were coexpressed in HEK293 cells, recovered by IKKβ immune precipitation, digested with either trypsin or pepsin to obtain a mixture of complete or partially digested peptides, and analyzed by TiO_2_-LC-MS/MS [29,30]. A total of 853 unique peptides was identified from 6551 independent spectra, representing 89% coverage of the total protein sequence. All IKKβ peptides recovered by mass spectrometry including non-phosphorylated peptides are shown in Table S4 and Figure S2.

The frequency with which different phosphorylation sites were detected, termed spectral counting, is a widely accepted method of label-free quantification [36,37] and examination of the spectral counts associated with specific sites can be informative as discussed below. Although spectral counts of different peptides are not strictly comparable, large differences are well known to generally correlate with differences in relative abundance despite inherent limitations arising from differing ionization efficiencies of different peptides, or when combining results from different sample preparations and different proteases. Other factors serve to increase the confidence in the identification of a specific phosphorylation site, for example, detection of a specific phosphoamino acid on peptides generated by two different proteases, or identification of a phosphoamino acid on a peptide with only a single S/T/Y hydroxyl amino acid phosphoacceptor site within the peptide.

Despite the typically low abundance of Tyr phosphorylation versus Ser phosphorylation, six Tyr residues were identified as phosphorylated (Table 1). The spectral counts of the 5 major phospho-Tyr residues are shown in Figure 2B, revealing that Tyr169 was the most frequently identified site of Tyr phosphorylation under our experimental conditions. A spectrum providing unequivocal identification of phospho-Tyr169 is presented in Figure 2D. The identification of phospho-Tyr169 was unambiguous given that this modification was identified in four separate sample preparations and on peptides generated both by trypsin (IIDLGY[243]AK) and pepsin (GY[243]AKELDQGSL; Table 1) and, furthermore, noting that Tyr169 is the sole hydroxyl amino acid on the tryptic peptide (IIDLGY[243]AK). The entirety of the IKKβ amino acid sequence is presented in Figure S2, showing all peptides and phosphopeptides detected in relation to structural motifs identified by crystallography [35]. Figure S2 readily reveals which areas of the protein yielded dense data defined by multiple overlapping peptides, versus the 11% of the protein that was not experimentally interrogated. 

**Table 1:** Tyrosine Phosphorylation of IKKβ: Analysis of Peptide Data.

**AA #**	**Prep #**	**Precursor Ion Charge**	**Peptide Sequence**	**Adjusted Probability**	**Spectral Count**	**Total Spectral Count**
**Y169**	#4899	2	IIDLGY[243]AK	0.9993	3	18 [39%]
	#4993	2	IIDLGY[243]AK	0.999	9	
	#4991	2	GY[243]AKELDQGSL	0.998	4	
	#4882	2	IIDLGY[243]AK	0.9969	2	
Y199*	#4991	2	LEQQKY[243]TVTVDY	0.0167	1	1 [2%]
**Y261**	#4899	2	FSSSLPY[243]PNNLNSVLAER	0.9999	3	7 [15%]
	#4882	3	FSSSLPY[243]PNNLNSVLAER	0.1521	1	
	#5038	2	SSSLPY[243]PNNL	0.1344	3	
**Y325**	#5038	2	VTGTIHTY[243]PVTEDESL	0.3137	3	7 [15%]
	#4991	2	M[147]VTGTIHTY[243]PVTEDESL	0.2753	2	
	#4991	2	VTGTIHTY[243]PVTEDESL	0.1539	2	
**Y397**	#4899	3	ITY[243]ETQISPRPQPESVSCILQEPK	0.9999	1	10 [22%]
	#4882	2	ITY[243]ETQISPR	0.9893	4	
	#4899	2	ITY[243]ETQISPR	0.9874	1	
	#4993	2	ITY[243]ETQISPR	0.0455	3	
	#4898	2	ITY[243]ETQISPR	0.0273	1	
**Y609**	#4993	2	VIY[243]TQLSK	0.9963	2	3 [7%]
	#4882	2	VIY[243]TQLSK	0.991	1	

All IKKβ peptides containing phospho-Tyr identified by TiO_2_-LC-MS/MS are presented here together with the peptide sequence, precursor ion charge, and the spectral count of each peptide. These results were obtained by analysis of 6 different samples prepared from HEK293 cells expressing FGFR2 and IKKβ, digested with either trypsin or pepsin (see Table S1). Within the peptide sequence, **Y**[243] indicates the presence of a pTyr residue as revealed by the mass of 243 Da (~163 Da for tyrosine plus ~80 Da for HPO_3_). Although many factors affect the recovery of individual peptides, the number of times each ion was detected in each preparation is indicated by the spectral count, which represents a widely accepted method of label-free quantification [36,37]. The last column indicates the total spectral count for each phospho-Tyr residue summed over all preps and, within square brackets, the proportion of the total phospho-Tyr-containing spectra. Note that 46 total phospho-Tyr spectra are summarized in Table 1, compared with 6551 total spectra (Table S1); thus, phospho-Tyr containing peptides represent 0.7% of the total peptides analyzed in this study. Information presented in this table represents a subset of information presented in Table S4, which presents the total analysis of IKKβ-derived peptides. **ADDITIONAL NOTES**: Peptides with nsp probability < 0.01 discarded; nsp refers to number of sibling peptides; **M**[147] indicates peptide containing oxidized Met residue; * indicates a low abundance precursor, only a single MS/MS spectrum, manually validated, second matching MS scan detected, but ambiguous assignment of Y199 with T200 and T202.

Mass spectrometry data also identified 9 sites of Thr phosphorylation (Table S2, ). The spectral counts of the 4 major phospho-Thr residues, T23, T180, T559, and T735, are shown in Figure 2C, again recognizing the limitations of spectral counting noted previously. A spectrum identifying phospho-Thr180 is presented in Figure 2E. Despite our demonstration of Tyr and Thr phosphorylation sites, the vast majority of phosphopeptides contained phospho-Ser residues, as shown in Tables S3-S5. Prior work has identified the importance of Ser phosphorylation within the C-terminal domain as a negative regulatory modification that contributes to a decrease in IKKβ activity, and peptide mapping directly demonstrated phosphorylation of Ser670 and Ser672 [24]. We detected phosphorylation on 8 of these C-terminal Ser residues, and that 3 of these (Ser672, Ser697, Ser733) are among the most abundantly phosphorylated residues with over 100 spectral counts each (Table S5). The other three most abundant sites of Ser phosphorylation observed in our study are Ser335 and Ser402 in the ubiquitin-like domain (ULD) and Ser634 in the Helix-loop-helix (HLH) domain. These sites may be relevant to the regulatory role of the ULD in osteoclastogenesis and osteolysis [38]. Phosphorylation of Ser257 within the kinase domain was observed in a recent structure of IKKβ [39], which we also observed (Table S5). 

**Figure 3:**
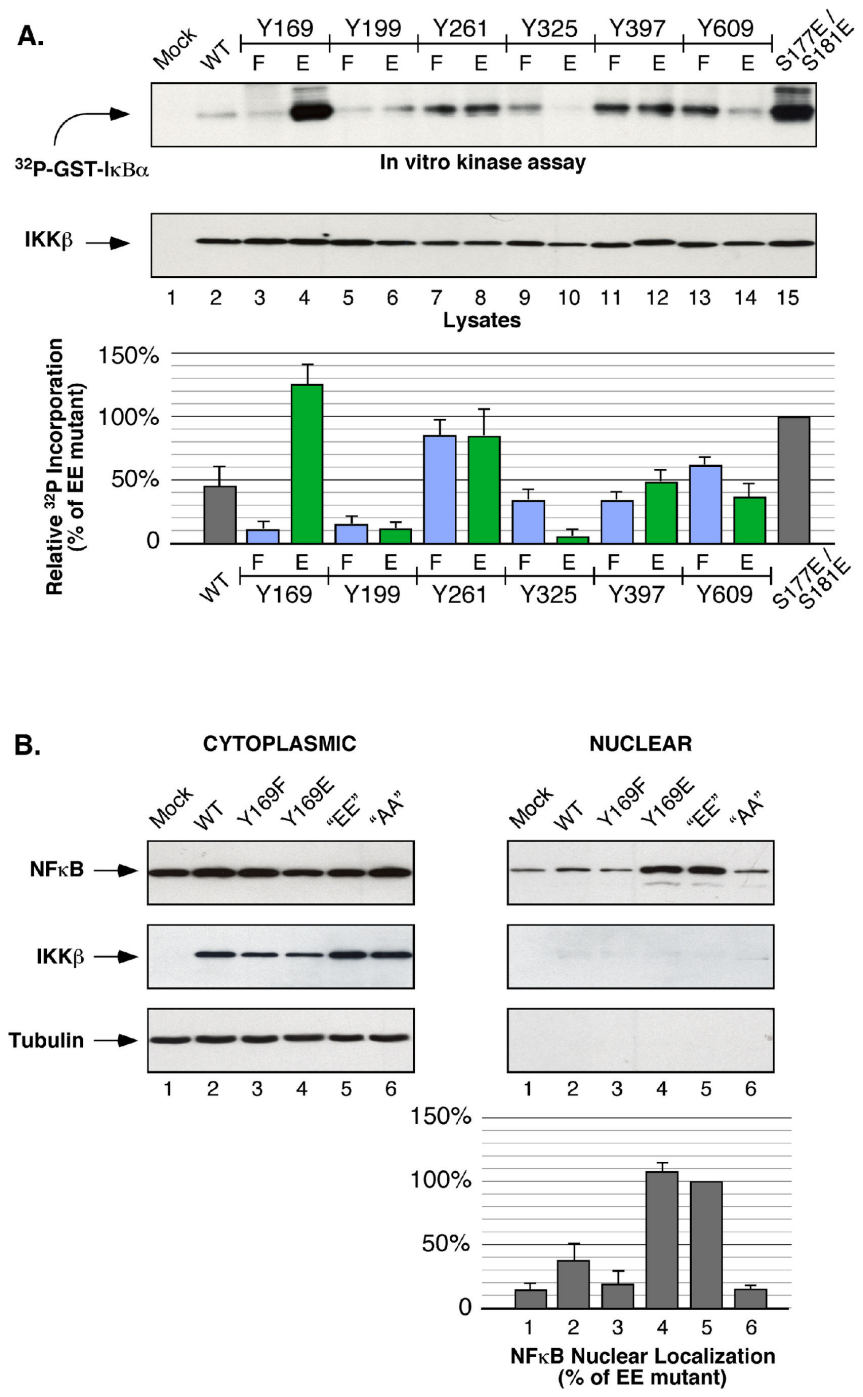
Effect of tyrosine mutations on IKKβ activation and NFκB nuclear localization. (**A**) **Effects on kinase activity of mutations at sites of IKKβ tyrosine phosphorylation**. Each tyrosine residue detected as phosphorylated by MS/MS analysis (see Table 1) was mutated either to phenylalanine (F) to prevent phosphorylation, or to glutamic acid (E) as the phosphomimic. Indicated mutants were expressed in HEK293 cells, and resulting IKKγ/NEMO immunoprecipitates were subjected to *in* *vitro* kinase assays utilizing the substrate GST-IκBα^(1-54)^ as described in Materials and Methods. ^32^P incorporation on GST-IκBα^(1-54)^ (top panel) and expression of IKKβ mutant proteins in cell lysates is shown (lower panel). Quantification of the relative ^32^P incorporation on GST-IκBα^(1-54)^ is presented in the bar graph below, showing the standard error of the mean for a minimum of 3 independent repeats, and normalized to the activity of the S177E/S181E “EE” mutant as 100%. All IKKβ mutants presented here were untagged. (**B**) **Y169E promotes NFκB nuclear localization**. NFκB nuclear localization in response to IKKβ mutants. Indicated IKKβ proteins were expressed in MCF7 cells. Cells were fractionated as described in Materials and Methods. Cytoplasmic and Nuclear lysate fractions were immunoblotted for NFκB (p65) (top panels) and β-Tubulin (bottom panels). The top membrane was stripped and reprobed for expression of IKKβ (middle panels). Quantification of NFκB nuclear localization is presented in the bar graph below, showing the standard error of the mean for a minimum of 3 independent repeats, normalized to the activity of the S177E/S181E “EE” mutant as 100%.

Figure 3A presents an *in vitro* kinase assay in which IKKγ/NEMO immune complexes were assayed using GST-IκBα ^(1-54)^ as the substrate [40], examining all Tyr residues that we identified as phosphorylated (Table 1) after mutation to Phe or Glu, the former to prevent phosphorylation and the latter as the phosphomimic, in comparison with the activated “EE” mutant, S177E/S181E. Most F/E mutants exhibited similar levels of kinase activation with several notable exceptions (Figure 3A). Significantly, the phosphomimic at Tyr169 within the IKKβ Activation Loop, Y169E, showed a level of kinase activation repeatedly equal to or slightly greater than the “EE” mutant. This suggested that phosphorylation at Tyr169 may provide an alternative mechanism of regulation of NFκB activation. 

Figure 3B presents the results of NFκB activation experiments examining the ability of selected IKKβ mutants to stimulate nuclear localization of endogenous NFκB. In comparison with the “EE” mutant, set to 100%, the phosphomimic Y169E stimulated equivalent nuclear NFκB localization. This result confirms that Y169E is able to stimulate significant IKKβ activation as evidenced by nuclear localization of the transcription factor NFκB. 

**Figure 4:**
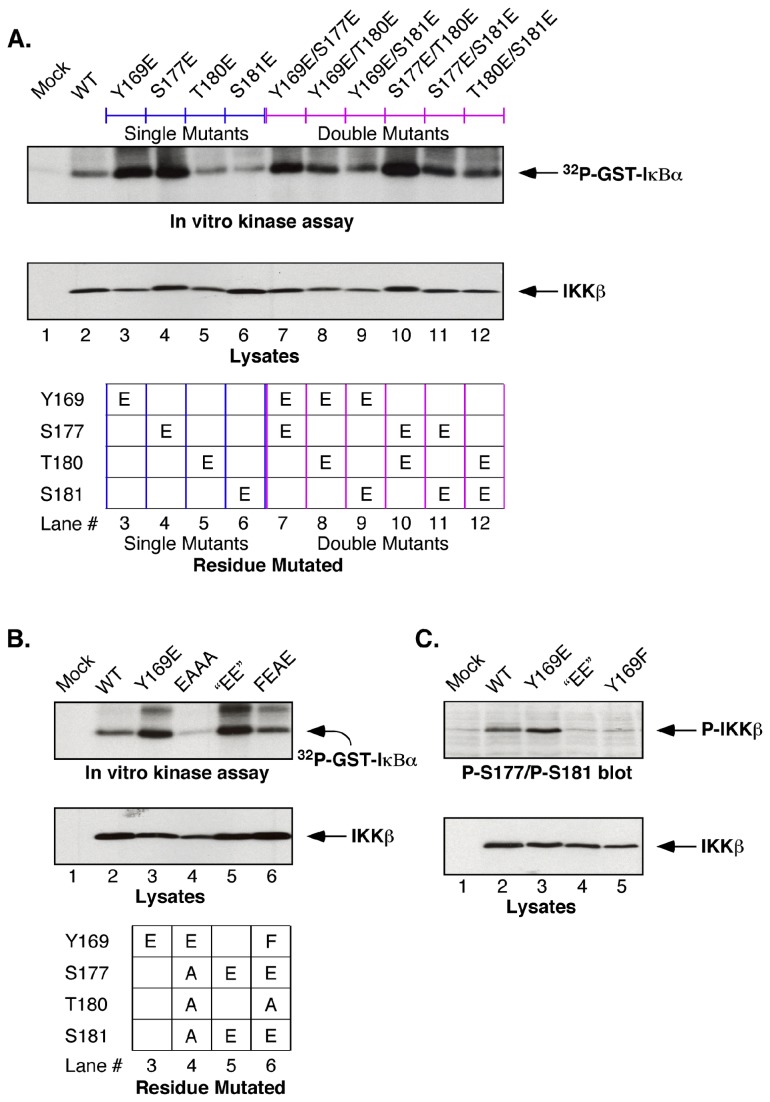
Composite analysis of mutations of phospho-acceptor sites within the IKKβ ***Acitvation*** ***Loop***. (**A**) **Contribution of Tyr169, Ser177, Thr180, and Ser181 to IKKβ kinase activation**. All possible combinations of single and double mutations were constructed in the IKKβ Activation Loop phospho-acceptor sites, Ser177 and Ser 181, identified previously [24], and Tyr169 and Thr180 identified in this work. Immunoprecipitated IKKγ/NEMO complexes from HEK293 cells were assayed for *in* *vitro* kinase activity against the substrate GST-IκBα^(1-54)^ (top panel), and IKKβ expression is shown (lower panel). (**B**) **Requirement for multiple hydroxyl amino acids within Activation Loop**. Multiple mutations within the Activation Loop probe minimal requirements for activation. Mutations were constructed within the Activation Loop phospho-acceptor sites to examine whether Y169E could provide activation when combined with the mutations S177A, T180A, and S181A (compare Lanes 3 and 4). Similarly, Lanes 5 and 6 examine the ability of the “EE” mutations S177E/S181E to provide activation when combined with Y169F and T180A. IKKγ/NEMO immunoprecipiates were examined for *in* *vitro* kinase activity against the substrate GST-IκBα^(1-54)^ (top panel). IKKβ expression is shown (lower panel). (**C**) **Y169E stimulates S177/S181 phosphorylation**. Activation Loop phosphorylation detected using phospho-S177/S181 antiserum. The ability of IKKβ WT and Y169F to stimulate phosphorylation of S177/S181 as detected by phospho-specific immunoblotting is presented, in comparison with the lack of activity shown by the S177E/S181E “EE” and Y169F mutants. IKKβ expression is shown (lower panel).

Having identified Tyr169 as a site of Tyr phosphorylation stimulated by FGFR2, we examined the contribution of each of the hydroxyl amino acids within the IKKβ Activation Loop for a fully activated kinase. Using *in vitro* kinase assays of IKKγ/NEMO immune complexes, against the substrate GST-IκBα^(1-54)^ as before, Figure 4A shows the ^32^P-incorporation for all possible single and double mutants at Tyr169, Ser177, Thr180, and Ser181. Surprisingly, among the single mutants, Y169E provided a level of activation comparable to S177E, and greater than T180E or S181E. Among all 6 possible double mutants, the mutants Y169E/S177E, Y169E/T180E, and S177E/T180E showed the greatest activation, with a lower level of activation exhibited by the other double mutants including the “EE” mutant. 

We also analyzed the ability of Y169E to activate IKKβ (Figure 4B) in the presence (Lane 3) or absence (Lane 4) of the other 3 hydroxyl amino acids within the Activation Loop, Ser177, Thr180, and Ser181. These results show clearly that one or more of these other hydroxyl amino acids is required, presumably to stabilize an activated conformation [35] resulting from Y169E. Conversely, Lane 6 shows that the activated “EE” mutant loses activity when combined with the mutations Y169F and T180A, again demonstrating a requirement for other hydroxyl amino acid residues within the Activation Loop to stabilize an active conformation.

Using an antiserum specific for detection of phospho-S177/phospho-S181 [41,42], we probed whether the activated IKKβ Y169E would stimulate Ser phosphorylation at either of these residues. Interestingly, a signal was observed indicating phosphorylation at S177/S181 in response to the Y169E mutant (Figure 4C, Lane 3). The observed phosphorylation of the IKKβ WT protein detected by this antiserum (Figure 4C, Lane 2) is consistent with other reports of basal autoactivation through oligomerization-dependent trans auto-phosphorylation [33].

We also considered the possibility that phosphorylation at Tyr169 may require prior phosphorylation at one of the other hydroxyl amino acids Ser177, Thr180, or Ser181. In this case, we expected to recover doubly phosphorylated peptides containing phospho-Tyr169. No such peptides were identified (Table 1), in spite of our ability to detect doubly and triply phosphorylated peptides (Tables S2 and S3). Additionally, under the experimental conditions utilized here, we detected no phosphorylation of Ser177 and only minor phosphorylation of Ser181 (Table S5).

## Discussion

Two IKKβ crystal structures have been obtained with the Glu phosphomimic activating mutations S177E/S181E, whereas a recent structure has been solved with the actual phospho groups at these residues [33,35,39]. In the active protein structure, Tyr169 is located on the surface of IKKβ exposed to the solvent interface, being readily accessible for phosphorylation by an interacting protein. Moreover, in the inactive structure, Tyr169 is located in a highly dynamic region within the activation loop. Therefore, phosphorylation at Tyr169 in the inactive wild-type protein certainly has the potential to result in a conformational change similar to that reported for the S177E/S181E mutant. 

The mass spectrometry data clearly show that in the presence of FGFR2, activated by overexpression in HEK293 cells, IKKβ undergoes significant phosphorylation revealing a rich resource of potentially important phosphorylation sites. Some of these phosphorylations, such as the Tyrosine phosphorylations, are clearly due to the presence of FGFR2 since phospho-Tyr can not be detected on IKKβ in the absence of FGFR2 (Figure 1D, Lane 4); nonetheless, it is unclear if the phospho-Ser and phospho-Thr sites identified are uniquely the result of FGFR2 expression. Some phosphorylation sites have been previously discovered, as in seminal work identifying Ser177/Ser181 within the Activation Loop and a cluster of Ser phosphorylation sites within the C-terminal domain that appear to inhibit signaling [24,43]. Within the Activation Loop, we have identified two novel sites of phosphorylation: Tyr169 as a principal site of Tyr phosphorylation; and Thr180, which was a relatively abundant site of Thr phosphorylation. Phosphorylation at Thr735 most likely contributes to the C-terminal negative regulation, whereas phosphorylation at Thr23 is likely to be of interest as a site of potential regulation, lying within the Region 1 GxGxxG of the ATP-binding domain and thus analogous to the phosphorylations that inhibit Cdk2 and related cyclin-dependent kinases [44]. 

With regard to Tyr phosphorylation, RTKs constitute the second largest category of cell surface receptors after the G-protein coupled family [45]. Crosstalk between RTKs and NFκB signaling has been reported by largely indirect mechanisms [46-50]. Perhaps not surprisingly, in light of the central importance of NFκB signaling, our work shows clearly that IKKβ can directly integrate signals resulting from Tyr and Thr phosphorylation, as well as the much studied Ser phosphorylation. Given the importance of many RTKs to human development and disease, and the nearly ubiquitous role played by NFκB signaling, the experiments described here suggest the existence of novel signaling connections between RTKs and NFκB regulatory pathways. 

We have previously observed that FGFR4 signaling attenuates TNFα stimulated NFκB signaling [31], as also recently reported for FGFR2 [51]. This latter work [51] demonstrated that FGFR2 serves as a scaffold for regulation of NFκB signaling and showed, much as is the case for FGFR4 [31], that FGFR2 expression reduces STAT3 phosphorylation, nuclear RelA/p65 NFκB translocation, and expression of NFκB-dependent transcripts such as interleukin-6, leading overall to reduced breast cancer cell proliferation/invasiveness. Our results here that FGFR2 activation induces phosphorylation of Tyr169, together with the fact that the Y169E phosphomimetic confers strong activation of IKKβ kinase activity, might initially appear contradictory. Two explanations may be useful to consider: first, the phosphorylation of Tyr169 is in fact accompanied by many other phosphorylation events, the combined result of which may be inhibitory rather than stimulatory. It will require detailed studies to understand the regulatory importance, for example, of the phosphorylations reported here at Thr23 within the Region 1 ATP-binding domain, or at Tyr397 between the Ubiquitin-Like Domain and the Scaffold/Dimerization Domain, just to mention two potentially interesting sites. A second explanation may be that activation of IKKβ via Activation Loop tyrosine phosphorylation,whether this occurs directly by FGFR2 or some other protein tyrosine kinase, may trigger a greater initial burst of NFκB-dependent inflammatory responses, but might also trigger a more robust cascade of C-terminal inhibitory Ser/Thr phosphorylations leading to more rapid attenuation of activated IKKβ and a more pronounced downregulation of cytokine-induce signaling.

The importance of IKKβ as a clinical target for the development of small molecule pharmacological inhibitors has resulted in the development of several potentially efficacious drugs [52-55]. These efforts have been primarily targeted against the active conformation represented by the standard “EE” S177E/S181E double mutant, as revealed by high resolution structure determinations (3QA8, 4E3C, 4KIK) [33,35,39]. The fact that IKKβ activation can be achieved by alternative phosphorylation events as described here, distinct from the canonical NFκB pathway, suggests that a larger universe of activated conformations will need to be considered to achieve optimal inhibition. 

## Materials and Methods

### Cell Culture

HEK293 cells were grown in DMEM with 10% FBS and 1% Pen/Strep and maintained in 10% CO_2_ at 37°C. Cells were transfected with plasmid DNA using calcium phosphate precipitation at 3% CO_2_, as previously described [56]. MCF7 cells were grown in DMEM with 10% FBS and 1% Pen/Strep and maintained in 5% CO_2_ at 37°C. MCF7 cells were transfected with Lipofectamine 2000 per manufacturer’s directions.

### FGFR2 and IKKβ constructs

FGFR2 [57] and IKKβ [31] expression plasmids have been described previously. FGFR2 kinase dead (K517R) and all mutations in IKKβ were created by Quikchange site-directed mutagenesis and confirmed by DNA sequencing. The GST-IκBα ^(1-54)^ plasmid used to generate the bacterially expressed substrate was provided by Prof. Alexander Hoffmann (UCSD). The C-terminus of IKKβ (aa 560-756) was subcloned into the pMAL-C2E vector at BamHI and NotI sites to create the MBP-HLH+ fusion protein. Fragments of the intracellular domain of FGFR2 were subcloned into pGEX6P using EcoRI and XbaI sites. [GST-JMD (aa 399-489), GST-KD1 (aa 489-581), GST-KD1+ (aa 489-598), GST-KD2 (aa 598-757), GST-Cterm (aa 758-822)]. 

### Antibodies, immunoprecipitation and immunoblotting

Antibodies were obtained from the following sources: Bek/FGFR2 (C-17), IKKβ (H-4), IKKγ (FL-419), NFκB p65 (F-6), β-tubulin (H-235), GST (Z-5) from Santa Cruz Biotechnology; 4G10 (antiphosphotyrosine) from Upstate Biotechnology; Phospho-IKKα/β (Ser176/180) from Cell Signaling; horseradish peroxidase (HRP) anti-mouse, HRP anti-rabbit from GE Healthcare. The enhanced chemiluminence (ECL) reagents were from GE Healthcare. HEK293 cells were transfected and starved as described [58]. Coimmunopreciptations and immunoblotting experiments were carried out using previously described procedures [58]. All immunoblotting experiments were repeated a minimum of 3 times, even those for which quantification is not presented.

### Mass spetrometry analysis

HEK293 cells were plated one day prior to transfection at 3.0 x 10^6^ cells per 15cm tissue culture plate. 10 plates were transfected with FGFR2 and IKKβ expression plasmids as described [58]. Cells were washed once in 1xPBS + 1mM Na_3_VO_4_ before being lysed in RIPA (50mM Tris pH 7.5, 150mM NaCl, 1% Triton X-100, 1% DOC, 0.1% SDS, 50mM NaF, 0.1mM PMSF, 10μg/ml Aprotinin, 1mM Na_3_VO_4_). Clarified lysates were immunoprecipitated with IKKβ antisera overnight at 4°C with rocking. Protein A-Sepharose was added for 4 hr at 4°C with rocking to collect immune complexes. Samples were washed 3x with RIPA buffer and 3x with Wash Buffer (20mM Tris pH 7.4, 120mM NaCl, 1mM Na_3_VO_4_, 20mM β-glycerophosphate). Samples were then treated with Elution Buffer (100mM NH_4_HCO_3_ pH 8.3, 8M Urea, 10mM DTT) for 20 min at 65°C to reduce the disulfide bonds which also eluted proteins from the sepharose beads. Proteins were alkylated with 10mM iodoacetamide for 10 min at room temperature. Urea was diluted to less than 2M with 20mM HEPES pH 8.0. Samples were digested with immobilized TPCK-trypsin (Thermo Scientific #20230) or with immobilized pepsin (Thermo Scientific # 20343) as indicated in Table S1, and incubated at room temperature with gentle rocking. Long (240 min) and short (5 min) digestions were performed. Long and short digestions were combined and protein A beads plus immobilized protease beads were removed from liquid using BioRad Poly-Prep Chromatography Column prior to desalting peptides using Waters (WAT 023590) C18 columns. Peptides were loaded on the column 4 times, washed with 5% acetonitrile / 5% acetic acid and eluted with 80% acetonitrile / 5% acetic acid. Peptides were lyophylized, resuspended in 70% acetonitrile / 0.5% NH_4_OH and dried again. Samples were analyzed by titanium dioxide-based phosphopeptide enrichment (TiO_2_)-liquid chromatography (LC)-tandem mass spectrometry (MS/MS) [29,30].

### In vitro kinase assays

HEK293 cells were transfected and starved overnight prior to lysing in Cytoplasmic Extract Buffer (10mM Hepes pH 7.9, 250mM NaCl, 0.5% NP-40, 1mM EDTA, 0.2% Tween-20, 1mM DTT, 1mM PMSF, 10mM NaF, 0.1mM Na_3_VO_4_, 20mM β-glycerophosphate). Lysates were immunoprecipitated with IKKγ anitsera, collected on Protein A-Sepharose, washed with Cytoplasmic Extract Buffer and Wash Buffer (20mM Hepes pH 7.9, 100mM NaCl, 10mM MgCl_2_, 2mM DTT, 1mM PMSF, 10mM NaF, 0.1mM Na_3_VO_4_, 20mM β-glycerophosphate) and subjected to *in vitro* kinase assay utilizing GST-IκBα^(1-54)^ as substrate. Kinase reactions containing 10 μCi of [γ-^32^P]-ATP supplemented with 20 μM ATP, and 0.5 μg of purified GST-IκBα^(1-54)^ were incubated at 30°C for 30 min, and separated by 12.5% SDS-PAGE. Gels were stained, destained, dried and exposed to film. All kinase assays were repeated a minimum of 3 times, even those for which quantification is not presented. Where shown, quantification of a minimum of three replicate experiments is presented normalized against IKKβ S177E/S181E “EE” mutant, which was set at 100%. Error bars show standard error of the mean. 

### In vitro binding assays

Plasmids containing MBP-HLH+ and GST-FGFR2 fusion domains were transformed into Rosetta cells. Single colonies were grown to A_600_ 0.4-0.6 prior to induction with 0.1M IPTG for 3 hr at 37°C. Bacteria were pelleted, resuspended in 1xPBS with 2mM EDTA, 1mM DTT, 1mM PMSF and sonicated. The GST-FGFR2 proteins were purified with glutathione-agarose beads while MPB-HLH+ was purified with amylose resin (NEB). Purified proteins were combined in Binding Buffer (50mM Tris pH 7.5, 100mM NaCl, 0.1mM EDTA, 0.5mM DTT) and allowed to incubate with rotation overnight at 4°C. Amylose resin was added, samples incubated with rotation at 4°C for 2 hr then washed three times with Binding Buffer. Proteins were eluted with 10mM maltose in Binding Buffer, separated by 12.5% SDS-PAGE and transferred to Immobilon-P membranes for immunoblotting. 

### NFκB localization by cell fractionation

MCF7 cells were transfected with Lipofectamine 2000 with no serum or antibiotics in media. Cells were collected 20 hr later for fractionation. Cell pellets were resuspended in CE Buffer (10mM Hepes pH 7.9, 60mM KCl, 1mM EDTA, 0.5% NP-40, 1mM DTT, 1mM PMSF). After vortexing and pelleting the cytoplasmic lysate was transferred to new tubes. Nuclear pellets were washed twice with CE Buffer before adding NE Buffer (250mM Tris pH7.5, 60mM KCl, 1mM EDTA, 1mM DTT, 1mM PMSF). Pellets were frozen on dry ice and thawed three times. Nuclear extracts were collected by centrifugation. Protein concentrations were determined by Bradford Assay. Equal amounts of total protein were separated by 15% SDS-PAGE and transferred to Immobilon-P membrane for immunoblotting.

## Supporting Information

Figure S1 ***Invitro* binding of FGFR2 and IKKβ.** *In* *vitro* binding assay determines the domain of FGFR2 which interacts with IKKβ. (**A**) A schematic of FGFR2 is shown. The extracellular domain contains three Ig-like regions (Ig) followed by the transmembrane domain (TM). The intracellular regions were isolated into domains: the juxtamembrane (JM), the 1st and 2nd kinase (KD1 and KD2) and the C terminal (Cterm) domains, each of which were fused in frame to glutathione-S-transferase (GST). The amino acids of FGFR2 that were used in the fusion proteins are indicated. (**B**) A schematic of IKKβ is shown with the N-terminal kinase domain (KD), the ubiqutin-like domain (ULD), the scaffold/dimerization domain (SDD) which also contains the leucine zipper (LZ) and helix-loop-helix (HLH) regions. The NEMO binding domain (NBD) is at the C-terminus of IKKβ. The region of IKKβ that was used in the MBP fusion protein, which includes HLH is indicated. (**C**) GST-FGFR2 and MBP-HLH+ fusion constructs were bacterially expressed and purified as described in Materials and Methods. The purified MBP-HLH+ protein was incubated with the GST-FGFR2 fusion proteins overnight and complexes were recovered with amylose resin. After washing, the proteins that bound to MBP-HLH+ were eluted with buffer containing maltose. Samples of the input GST-FGFR2 fusion proteins and the elutions were separated by 12.5% SDS-PAGE, transferred to Immobilon-P membrane and immunoblotted with anti-GST sera. Controls for the nonspecific binding of the GST-KD1+ and GST-KD2 proteins to the amylose resin were performed and only showed binding in the presence of MBP-HLH+ protein (data not shown).
(PDF)

Figure S2 **Location of All Unique IKKβ Peptides and Phosphopeptides Identified.** The IKKβ amino acid sequence is shown together with the location of all identified peptides and phosphopeptides with respect to structural motifs [35]. Boundaries were arbitrarily set for the relative abundance for P-Thr and P-Tyr sites at: <5% of total scans, RARELY OBSERVED SITE; 5-15% of total scans, INTERMEDIATE SITE; ≥ 15% of total scans, ABUNDANT SITE. Due to the large number of P-Ser scans, boundaries were arbitrarily set for the relative abundance for P-Ser sites at: <1% of total scans, RARELY OBSERVED SITE; 1-9% of total scans, INTERMEDIATE SITE; ≥ 9% of total scans, ABUNDANT SITE.
(PDF)

Table S1 **Description of Protein Samples Used for Mass Spectrometry.**
(PDF)

Table S2 **Threonine Phosphorylation of IKKβ: Analysis of Peptide Data.**
(PDF)

Table S3 **Serine Phosphorylation of IKKβ: Analysis of Peptide Data.**
(PDF)

Table S4 **Peptide Analysis of IKKβ: Total Peptides.**
(PDF)

Table S5 **Serine Phosphorylation Sites by Spectral Counts.**
(PDF)
