# Supplementary figures and images for: Tyrosine Phosphorylation Allows Integration of Multiple Signaling Inputs by IKKβ
Source: PLoS One. 2013 Dec 27;8(12):e84497. doi: 10.1371/journal.pone.0084497 (PMC3873999; doi:10.1371/journal.pone.0084497)

Figure S1

A. FGFR2

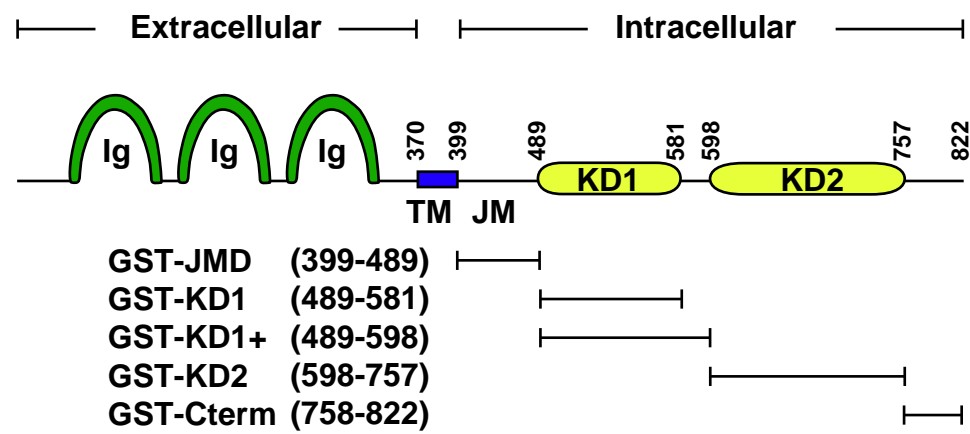

B. IKK $\beta$

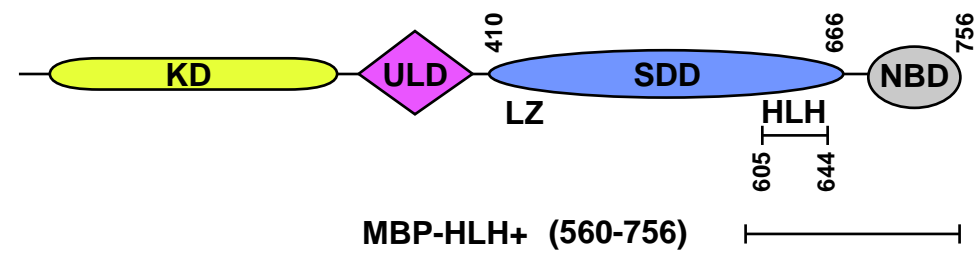

C.

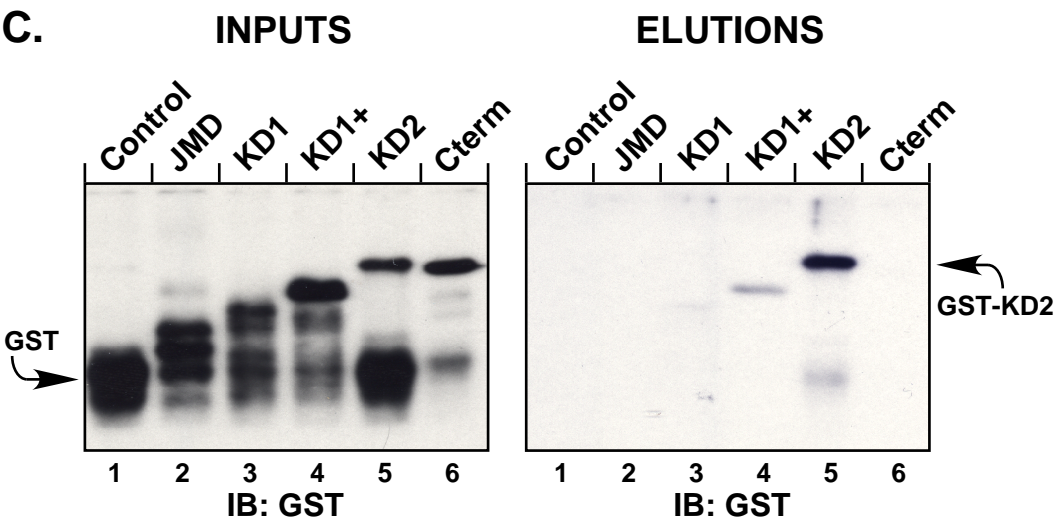

Supplement: Figure S1 — Invitro binding of FGFR2 and IKKβ. In vitro binding assay determines the domain of FGFR2 which interacts with IKKβ. (A) A schematic of FGFR2 is shown. The extracellular domain contains three Ig-like regions (Ig) followed by the transmembrane domain (TM). The intracellular regions were isolated into domains: the juxtamembrane (JM), the 1st and 2nd kinase (KD1 and KD2) and the C terminal (Cterm) domains, each of which were fused in frame to glutathione-S-transferase (GST). The amino acids of FGFR2 that were used in the fusion proteins are indicated. (B) A schematic of IKKβ is shown with the N-terminal kinase domain (KD), the ubiqutin-like domain (ULD), the scaffold/dimerization domain (SDD) which also contains the leucine zipper (LZ) and helix-loop-helix (HLH) regions. The NEMO binding domain (NBD) is at the C-terminus of IKKβ. The region of IKKβ that was used in the MBP fusion protein, which includes HLH is indicated. (C) GST-FGFR2 and MBP-HLH+ fusion constructs were bacterially expressed and purified as described in Materials and Methods. The purified MBP-HLH+ protein was incubated with the GST-FGFR2 fusion proteins overnight and complexes were recovered with amylose resin. After washing, the proteins that bound to MBP-HLH+ were eluted with buffer containing maltose. Samples of the input GST-FGFR2 fusion proteins and the elutions were separated by 12.5% SDS-PAGE, transferred to Immobilon-P membrane and immunoblotted with anti-GST sera. Controls for the nonspecific binding of the GST-KD1+ and GST-KD2 proteins to the amylose resin were performed and only showed binding in the presence of MBP-HLH+ protein (data not shown). (PDF) [file pone.0084497.s001.pdf]

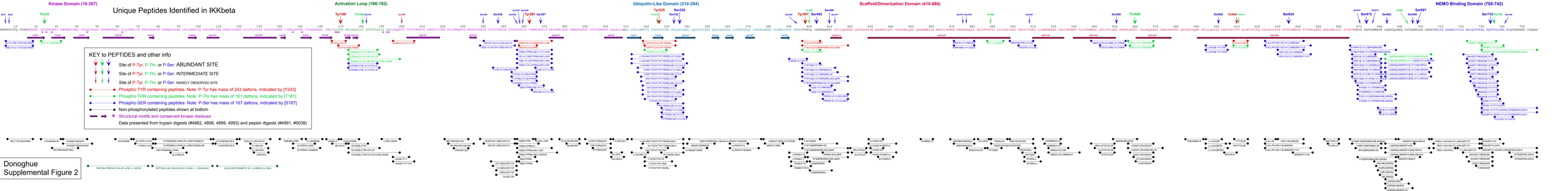

Supplement: Figure S2 — Location of All Unique IKKβ Peptides and Phosphopeptides Identified. The IKKβ amino acid sequence is shown together with the location of all identified peptides and phosphopeptides with respect to structural motifs [35]. Boundaries were arbitrarily set for the relative abundance for P-Thr and P-Tyr sites at: <5% of total scans, RARELY OBSERVED SITE; 5-15% of total scans, INTERMEDIATE SITE; ≥ 15% of total scans, ABUNDANT SITE. Due to the large number of P-Ser scans, boundaries were arbitrarily set for the relative abundance for P-Ser sites at: <1% of total scans, RARELY OBSERVED SITE; 1-9% of total scans, INTERMEDIATE SITE; ≥ 9% of total scans, ABUNDANT SITE. (PDF) [file pone.0084497.s002.pdf]
