# Supplementary material for: Tyrosine Phosphorylation Allows Integration of Multiple Signaling Inputs by IKKβ
Source: PLoS One. 2013 Dec 27;8(12):e84497. doi: 10.1371/journal.pone.0084497 (PMC3873999; doi:10.1371/journal.pone.0084497)
Supplement: Table S1 — Description of Protein Samples Used for Mass Spectrometry. (PDF) [file pone.0084497.s003.pdf]

Table S1: Description of IKKβ Samples Used for Mass Spec Analysis

PREP #	PROTEASE	IKKβ SEQUENCE COVERAGE	NUMBER OF PEPTIDES	NUMBER OF SPECTRA
#4882	Trypsin	74% coverage	143 unique peptides	1315 independent spectra
#4898	Trypsin	60% coverage	107 unique peptides	506 independent spectra
#4899	Trypsin	68% coverage	132 unique peptides	1203 independent spectra
#4991	Pepsin	36% coverage	71 unique peptides	470 independent spectra
#4993	Trypsin	83% coverage	270 unique peptides	2586 independent spectra
#5038	Pepsin	48% coverage	130 unique peptides	471 independent spectra
TOTAL		89% coverage	853 unique peptides	6551 independent spectra
