# Supplementary material for: Tyrosine Phosphorylation Allows Integration of Multiple Signaling Inputs by IKKβ
Source: PLoS One. 2013 Dec 27;8(12):e84497. doi: 10.1371/journal.pone.0084497 (PMC3873999; doi:10.1371/journal.pone.0084497)
Supplement: Table S2 — Threonine Phosphorylation of IKKβ: Analysis of Peptide Data. (PDF) [file pone.0084497.s004.pdf]

Table S2: Threonine Phosphorylation of IKKβ: Analysis of Peptide Data *(see notes at end)*

Residue	Prep #	Precursor Ion Charge	Peptide Sequence	Adjusted Probability	Spectral Count	Total Spectral Count
**T23**	#4882	2	LGT[181]GGFGNVIR	1	4	17 [15%]
	#4899	2	LGT[181]GGFGNVIR	0.9999	4	
	#4993	2	LGT[181]GGFGNVIR	0.9999	4	
	#4898	2	LGT[181]GGFGNVIR	0.9998	5	
**T180**	#4898	3	ELDQGSLCT[181]SFVGTLQYLAPELLEQQK	0.9999	2	10 [9%]
	#4993	3	ELDQGSLCT[181]SFVGTLQYLAPELLEQQK	0.9997	1	
	#4882	2	ELDQGSLCT[181]SFVGTLQY	0.9977	3	
	#4993	2	ELDQGSLCT[181]SFVGTLQY	0.9921	2	
	#4899	2	ELDQGSLCT[181]SFVGTLQY	0.0508	1	
	#4993	2	ELDQGSLCT[181]SFVGTLQ	0.0193	1	
**T324**	#5038	2	VTGTIHT[181]YPVTE	0.0361	1	1 [1%]
**T399**	#4899	2	ITYET[181]QISPR	0.1275	1	2 [2%]
	#4993	2	ITYET[181]QISPR	0.0309	1	
**T488**	#5038	2	FKT[181]SIQIDL	0.1484	2	3 [3%]
	#4991	2	FKT[181]SIQIDL	0.052	1	
**T559**	#4882	2	KQGGT[181]LDDLEEQAR	1	3	62 [54%]
	#4882	3	KQGGT[181]LDDLEEQAR_ETD	1	2	
	#4898	3	KQGGT[181]LDDLEEQAR_ETD	1	6	
	#4899	3	KQGGT[181]LDDLEEQAR_ETD	1	8	
	#4882	2	QGGT[181]LDDLEEQAR	1	4	
	#4898	2	KQGGT[181]LDDLEEQAR	0.9999	5	
	#4899	2	KQGGT[181]LDDLEEQAR	0.9999	5	
	#4993	2	KQGGT[181]LDDLEEQAR	0.9999	9	
	#4993	3	KQGGT[181]LDDLEEQAR_ETD	0.9999	7	
	#4898	2	QGGT[181]LDDLEEQAR	0.9999	5	
	#4993	2	QGGT[181]LDDLEEQAR	0.9999	4	
	#4899	2	QGGT[181]LDDLEEQAR	0.9998	4	
**T610**	#4882	2	VIYT[181]QLSK	0.9907	1	2 [2%]
	#4993	2	VIYT[181]QLSK	0.976	1	
**T693**	#4899	3	LSQPGQLMSQPST[181]ASNSLPEPAKK	0.9948	1	2 [2%]
	#4993	3	LSQPGQLM[147]SQPST[181]ASNS[167]LPEPAKK	0.1228	1	

**T735**	#4898	3	EQDQSFT[181]ALDWSWLQTEEEEHSCLEQAS	0.9997	2	
	#4882	2	EQDQSFT[181]ALDWS	0.9964	5	
	#4991	2	NAIQDTVREQDQSFT[181]A	0.9953	1	15 [13%]
	#4993	2	EQDQSFT[181]ALDWS	0.3951	4	
	#4898	2	EQDQSFT[181]ALDWS	0.3089	2	
	#4899	2	EQDQSFT[181]ALDWS	0.0624	1	

Notes:

T[181] indicates pThr residue in peptide.

S[167] indicates pSer residue in peptide.

M[147] indicates peptide containing oxidized Met residue.

_ETD indicates peptide identification via an Electron-Transfer Dissociation (ETD) MS/MS spectrum.

Peptides with nsp probability < 0.01 discarded.
