# Supplementary material for: Tyrosine Phosphorylation Allows Integration of Multiple Signaling Inputs by IKKβ
Source: PLoS One. 2013 Dec 27;8(12):e84497. doi: 10.1371/journal.pone.0084497 (PMC3873999; doi:10.1371/journal.pone.0084497)
Supplement: Table S3 — Serine Phosphorylation of IKKβ: Analysis of Peptide Data. (PDF) [file pone.0084497.s005.pdf]

## Table S3: Serine Phosphorylation of IKKβ: Analysis of Peptide Data *(see notes at end)*

| Residue | Prep # | Precursor Ion Charge | Peptide Sequence | Adjusted Probability | Spectral Count | Total Spectral Count |
|---|---|---|---|---|---|---|
| **S4** | #4993 | 2 | S[167]PSLTTQTCGAWEM[147]K | 0.9947 | 1 | 4 [<1%] |
| | #4898 | 2 | S[167]PSLTTQTCGAWEM[147]K | 0.9933 | 2 | |
| | #4993 | 2 | S[167]PSLTTQTCGAWEMK | 0.9892 | 1 | |
| **S6** | #4993 | 2 | SPS[167]LTTQTCGAWEM[147]K | 0.9857 | 2 | 3 [<1%] |
| | #4898 | 2 | SPS[167]LTTQTCGAWEM[147]K | 0.0355 | 1 | |
| **S181** | #4898 | 2 | ELDQGSLCTS[167]FVGTLQY | 0.9943 | 4 | 11 [1%] |
| | #4993 | 2 | ELDQGSLCTS[167]FVGTLQY | 0.9898 | 2 | |
| | #4899 | 2 | ELDQGSLCTS[167]FVGTLQY | 0.819 | 2 | |
| | #4993 | 2 | ELDQGSLCTS[167]FVGTLQ | 0.0587 | 1 | |
| | #4898 | 2 | ELDQGSLCTS[167]FVGTLQ | 0.0287 | 2 | |
| **S239** | #4993 | 3 | QKS[167]EVDIVVSEDLNGTVK | 0.1177 | 1 | 5 [<1%] |
| | #4898 | 3 | QKS[167]EVDIVVSEDLNGTVK | 0.1064 | 1 | |
| | #4882 | 3 | QKS[167]EVDIVVSEDLNGTVK | 0.0896 | 3 | |
| **S246** | #4882 | 2 | SEVDIVVS[167]EDLNGTVK | 1 | 4 | 21 [2%] |
| | #4898 | 2 | SEVDIVVS[167]EDLNGTVK | 1 | 4 | |
| | #4882 | 3 | SEVDIVVS[167]EDLNGTVK_ETD | 1 | 3 | |
| | #5038 | 2 | IVVS[167]EDLNGTVKF | 0.9999 | 5 | |
| | #4898 | 3 | SEVDIVVS[167]EDLNGTVK_ETD | 0.9999 | 1 | |
| | #4991 | 2 | IVVS[167]EDLNGTVKF | 0.9996 | 2 | |
| | #4899 | 2 | SEVDIVVS[167]EDLNGTVK | 0.9996 | 2 | |
| **S256** | #4993 | 2 | FS[167]SSLPYPNNLNSVLAER | 0.9999 | 2 | 5 [<1%] |
| | #4899 | 2 | FS[167]SSLPYPNNLNSVLAER | 0.9992 | 1 | |
| | #4898 | 2 | FS[167]SSLPYPNNLNSVLAER | 0.9986 | 1 | |
| | #5038 | 2 | TVKFS[167]SSLPYPNNL | 0.9747 | 1 | |
| **S257** | #4899 | 2 | FSS[167]SLPYPNNLNSVLAER | 1 | 2 | 12 [1%] |
| | #4993 | 2 | FSS[167]SLPYPNNLNSVLAER | 0.9999 | 1 | |
| | #4882 | 2 | FSS[167]SLPYPNNLNSVLAER | 0.9999 | 1 | |
| | #4898 | 2 | FSS[167]SLPYPNNLNSVLAER | 0.9998 | 1 | |
| | #5038 | 2 | TVKFSS[167]SLPYPNNL | 0.9948 | 5 | |
| | #4898 | 3 | FSS[167]SLPYPNNLNSVLAER | 0.3826 | 1 | |
| | #5038 | 2 | VKFSS[167]SLPYPNNL | 0.0203 | 1 | |

| | | | | | | |
|---|---|---|---|---|---|---|
| **S258** | #4899 | 2 | FSSS[167]LPYPNNLNSVLAER | 1 | 1 | |
| | #4882 | 2 | FSSS[167]LPYPNNLNSVLAER | 1 | 3 | |
| | #4993 | 2 | FSSS[167]LPYPNNLNSVLAER | 0.9999 | 1 | |
| | #4898 | 2 | FSSS[167]LPYPNNLNSVLAER | 0.9999 | 2 | |
| | #4898 | 3 | FSSS[167]LPYPNNLNSVLAER | 0.9998 | 1 | 16 [1%] |
| | #5038 | 2 | TVKFSSS[167]LPYPNNL | 0.9942 | 5 | |
| | #4882 | 3 | FSSS[167]LPYPNNLNSVLAER | 0.7885 | 1 | |
| | #5038 | 2 | VKFSSS[167]LPYPNNL | 0.0598 | 1 | |
| | #4991 | 2 | TVKFSSS[167]LPYPNNL | 0.0396 | 1 | |
| **S267** | #4899 | 2 | FSSSLPYPNNLNS[167]VLAER | 1 | 4 | |
| | #4899 | 3 | FSSSLPYPNNLNS[167]VLAER | 1 | 4 | |
| | #4882 | 2 | FSSSLPYPNNLNS[167]VLAER | 1 | 6 | |
| | #4993 | 2 | FSSSLPYPNNLNS[167]VLAER | 0.9999 | 4 | |
| | #4993 | 3 | FSSSLPYPNNLNS[167]VLAER | 0.9998 | 1 | |
| | #4882 | 3 | FSSSLPYPNNLNS[167]VLAER | 0.9987 | 1 | |
| | #5038 | 2 | SSSLPYPNNLNS[167]VL | 0.9922 | 4 | 41 [4%] |
| | #4882 | 2 | NLNS[167]VLAER | 0.992 | 4 | |
| | #4899 | 2 | NLNS[167]VLAER | 0.9895 | 2 | |
| | #4991 | 2 | SSSLPYPNNLNS[167]VL | 0.9891 | 4 | |
| | #4898 | 2 | NLNS[167]VLAER | 0.8969 | 3 | |
| | #4993 | 2 | NLNS[167]VLAER | 0.3159 | 3 | |
| | #4993 | 2 | PNNLNS[167]VLAER | 0.1369 | 1 | |
| **S332** | #4898 | 3 | LVHILNM[147]VTGTIHTYPVTEDES[167]LQSLK | 1 | 2 | |
| | #4882 | 3 | M[147]VTGTIHTYPVTEDES[167]LQSLK | 1 | 3 | |
| | #4898 | 3 | M[147]VTGTIHTYPVTEDES[167]LQSLK | 1 | 3 | |
| | #4991 | 2 | VTGTIHTYPVTEDES[167]L | 1 | 6 | |
| | #4993 | 3 | LVHILNM[147]VTGTIHTYPVTEDES[167]LQSLK | 0.9999 | 2 | |
| | #4993 | 3 | LVHILNMVTGTIHTYPVTEDES[167]LQSLK | 0.9999 | 2 | |
| | #4993 | 2 | MVTGTIHTYPVTEDES[167]LQSLK | 0.9999 | 3 | |
| | #5038 | 2 | NM[147]VTGTIHTYPVTEDES[167]L | 0.9999 | 1 | |
| | #5038 | 2 | VTGTIHTYPVTEDES[167]L | 0.9999 | 4 | |
| | #4898 | 4 | LVHILNM[147]VTGTIHTYPVTEDES[167]LQSLK_ETD | 0.9995 | 1 | |
| | #4882 | 3 | MVTGTIHTYPVTEDES[167]LQSLK | 0.9988 | 4 | |
| | #4898 | 3 | LVHILNMVTGTIHTYPVTEDES[167]LQSLK | 0.9985 | 1 | |
| | #4898 | 3 | ILNM[147]VTGTIHTYPVTEDES[167]LQSLK | 0.9976 | 1 | 56 [5%] |
| | #4993 | 3 | MVTGTIHTYPVTEDES[167]LQSLK | 0.9951 | 4 | |
| | #4993 | 2 | PVTEDES[167]LQSLK | 0.995 | 4 | |
| | #5038 | 2 | M[147]VTGTIHTYPVTEDES[167]L | 0.7892 | 3 | |
| | #4899 | 3 | MVTGTIHTYPVTEDES[167]LQSLK | 0.4076 | 3 | |
| | #4991 | 2 | M[147]VTGTIHTYPVTEDES[167]L | 0.2757 | 1 | |
| | #4898 | 3 | MVTGTIHTYPVTEDES[167]LQSLK | 0.1384 | 3 | |
| | #4899 | 2 | M[147]VTGTIHTYPVTEDES[167]LQSLK | 0.1117 | 1 | |

| | | | | | | |
|---|---|---|---|---|---|---|
| | #5038 | 2 | MVTGTIHTYPVTEDES[167]L | 0.0849 | 1 | |
| | #4993 | 3 | M[147]VTGTIHTYPVTEDES[167]LQSLK | 0.0701 | 1 | |
| | #4993 | 2 | M[147]VTGTIHTYPVTEDES[167]LQSLK | 0.055 | 1 | |
| | #4882 | 3 | ILNM[147]VTGTIHTYPVTEDES[167]LQSLK | 0.0116 | 1 | |

| | | | | | | |
|---|---|---|---|---|---|---|
| **S335** | #4898 | 3 | ILNM[147]VTGTIHTYPVTEDESLQS[167]LK | 1 | 4 | |
| | #4898 | 3 | ILNMVTGTIHTYPVTEDESLQS[167]LK | 1 | 2 | |
| | #4899 | 3 | LVHILNM[147]VTGTIHTYPVTEDESLQS[167]LK | 1 | 1 | |
| | #4898 | 3 | LVHILNM[147]VTGTIHTYPVTEDESLQS[167]LK | 1 | 3 | |
| | #4898 | 4 | LVHILNM[147]VTGTIHTYPVTEDESLQS[167]LK_ETD | 1 | 1 | |
| | #4898 | 3 | LVHILNMVTGTIHTYPVTEDESLQS[167]LK | 1 | 4 | |
| | #4899 | 3 | M[147]VTGTIHTYPVTEDESLQS[167]LK | 1 | 11 | |
| | #4899 | 3 | MVTGTIHTYPVTEDESLQS[167]LK | 1 | 11 | |
| | #4898 | 2 | MVTGTIHTYPVTEDESLQS[167]LK | 1 | 6 | |
| | #4898 | 3 | MVTGTIHTYPVTEDESLQS[167]LK | 1 | 7 | |
| | #4993 | 3 | LVHILNMVTGTIHTYPVTEDESLQS[167]LK | 0.9999 | 9 | |
| | #4899 | 3 | LVHILNMVTGTIHTYPVTEDESLQS[167]LK | 0.9999 | 1 | |
| | #4993 | 2 | MVTGTIHTYPVTEDESLQS[167]LK | 0.9999 | 6 | |
| | #4993 | 2 | PVTEDESLQS[167]LK | 0.9999 | 5 | |
| | #4993 | 3 | PVTEDESLQS[167]LKAR_ETD | 0.9999 | 3 | |
| | #4993 | 4 | LVHILNM[147]VTGTIHTYPVTEDESLQS[167]LK_ETD | 0.9998 | 6 | |
| | #4993 | 4 | LVHILNMVTGTIHTYPVTEDESLQS[167]LK_ETD | 0.9998 | 8 | |
| | #4882 | 2 | MVTGTIHTYPVTEDESLQS[167]LK | 0.9997 | 3 | |
| | #4898 | 4 | LVHILNMVTGTIHTYPVTEDESLQS[167]LK_ETD | 0.9996 | 2 | |
| | #4882 | 2 | M[147]VTGTIHTYPVTEDESLQS[167]LK | 0.9994 | 3 | |
| | #4882 | 3 | MVTGTIHTYPVTEDESLQS[167]LK | 0.9993 | 2 | |
| | #4993 | 3 | MVTGTIHTYPVTEDESLQS[167]LK | 0.9989 | 8 | 153 [14%] |
| | #4993 | 4 | LVHILNM[147]VTGTIHTYPVTEDESLQS[167]LKAR_ETD | 0.9979 | 1 | |
| | #4898 | 3 | M[147]VTGTIHTYPVTEDESLQS[167]LK | 0.9973 | 7 | |
| | #4899 | 2 | MVTGTIHTYPVTEDESLQS[167]LK | 0.9972 | 2 | |
| | #4993 | 2 | TYPVTEDESLQS[167]LK | 0.9967 | 1 | |
| | #4993 | 2 | GTIHTYPVTEDESLQS[167]LK | 0.9961 | 2 | |
| | #4899 | 2 | PVTEDESLQS[167]LK | 0.9954 | 3 | |
| | #4899 | 3 | ILNM[147]VTGTIHTYPVTEDESLQS[167]LK | 0.995 | 3 | |
| | #4882 | 2 | PVTEDESLQS[167]LK | 0.9931 | 4 | |
| | #4993 | 3 | LVHILNM[147]VTGTIHTYPVTEDESLQS[167]LK | 0.9915 | 6 | |
| | #4898 | 2 | PVTEDESLQS[167]LK | 0.9915 | 1 | |
| | #4993 | 3 | ILNM[147]VTGTIHTYPVTEDESLQS[167]LK | 0.9897 | 2 | |
| | #4993 | 3 | M[147]VTGTIHTYPVTEDESLQS[167]LK | 0.944 | 5 | |
| | #4993 | 3 | NM[147]VTGTIHTYPVTEDESLQS[167]LK | 0.9157 | 1 | |
| | #4898 | 2 | M[147]VTGTIHTYPVTEDESLQS[167]LK | 0.182 | 1 | |
| | #4899 | 2 | M[147]VTGTIHTYPVTEDESLQS[167]LK | 0.1418 | 1 | |
| | #4993 | 3 | ILNMVTGTIHTYPVTEDESLQS[167]LK | 0.0768 | 1 | |
| | #4993 | 2 | M[147]VTGTIHTYPVTEDESLQS[167]LK | 0.0507 | 1 | |
| | #4882 | 3 | M[147]VTGTIHTYPVTEDESLQS[167]LK | 0.05 | 2 | |

| | | | | | | |
|---|---|---|---|---|---|---|
| | #4899 | 3 | ILNMVTGTIHTYPVTEDESLQS[167]LK | 0.0449 | 1 | |
| | #4882 | 3 | ILNM[147]VTGTIHTYPVTEDESLQS[167]LK | 0.0356 | 2 | |
| **S393** | #4991 | 2 | LFDNS[167]KITYE | 0.1022 | 2 | 2 [<1%] |
| **S402** | #4898 | 2 | ITYETQIS[167]PRPQPESVSC | 1 | 4 | |
| | #4899 | 3 | ITYETQIS[167]PRPQPESVSCILQEPK | 1 | 7 | |
| | #4882 | 3 | ITYETQIS[167]PRPQPESVSCILQEPK | 1 | 3 | |
| | #4898 | 3 | ITYETQIS[167]PRPQPESVSCILQEPK | 1 | 4 | |
| | #4993 | 2 | ITYETQIS[167]PRPQPESVSC | 0.9999 | 4 | |
| | #4993 | 3 | ITYETQIS[167]PRPQPESVSCILQEPK | 0.9999 | 5 | |
| | #4882 | 2 | ITYETQIS[167]PR | 0.9997 | 12 | |
| | #4898 | 4 | ITYETQIS[167]PRPQPESVSCILQEPK_ETD | 0.9997 | 1 | |
| | #4899 | 2 | ITYETQIS[167]PR | 0.999 | 13 | |
| | #4991 | 2 | TQIS[167]PRPQPESV | 0.9985 | 8 | |
| | #4899 | 2 | ITYETQIS[167]PRPQPESVSC | 0.9984 | 4 | 125 [12%] |
| | #4898 | 2 | ITYETQIS[167]PR | 0.9982 | 15 | |
| | #5038 | 2 | TQIS[167]PRPQPESV | 0.9943 | 10 | |
| | #4993 | 2 | ITYETQIS[167]PR | 0.9929 | 8 | |
| | #4991 | 2 | TQIS[167]PRPQPESVS | 0.9884 | 8 | |
| | #4993 | 2 | ITYETQIS[167]PRPQPESVS | 0.9861 | 4 | |
| | #4993 | 4 | ITYETQIS[167]PRPQPESVSCILQEPK_ETD | 0.9831 | 1 | |
| | #5038 | 2 | TQIS[167]PRPQPESVS | 0.9713 | 8 | |
| | #4898 | 3 | ITYETQIS[167]PRPQPESVSC | 0.2247 | 2 | |
| | #4993 | 3 | ITYETQIS[167]PRPQPESVSC | 0.1294 | 3 | |
| | #4899 | 3 | ITYETQIS[167]PRPQPESVSC | 0.0296 | 1 | |
| **S409** | #4882 | 2 | PQPES[167]VSCILQEPK | 0.9987 | 4 | |
| | #4898 | 2 | PQPES[167]VSCILQEPK | 0.9973 | 4 | |
| | #4899 | 2 | PQPES[167]VSCILQEPK | 0.9964 | 4 | 18 [2%] |
| | #4899 | 3 | ITYETQISPRPQPES[167]VSCILQEPK | 0.9953 | 1 | |
| | #4993 | 2 | PQPES[167]VSCILQEPK | 0.9951 | 4 | |
| | #4898 | 3 | ITYETQISPRPQPES[167]VSCILQEPK | 0.9827 | 1 | |
| **S411** | #4899 | 2 | PQPESVS[167]CILQEPK | 0.9928 | 1 | 2 [<1%] |
| | #4993 | 2 | PQPESVS[167]CILQEPK | 0.9881 | 1 | |
| **S474** | #4993 | 2 | NSMAS[167]MSQQLK | 0.9998 | 4 | |
| | #4993 | 2 | NSMAS[167]M[147]SQQLK | 0.9797 | 1 | 6 [<1%] |
| | #4898 | 2 | NSMAS[167]MSQQLK | 0.8299 | 1 | |
| **S476** | #4993 | 2 | NSMASM[147]S[167]QQLK | 0.9911 | 1 | 2 [<1%] |
| | #4993 | 2 | NSMASMS[167]QQLK | 0.4202 | 1 | |

| | | | | | | |
|---|---|---|---|---|---|---|
| **S507** | #4882 | 2 | YSEQTEFGITS[167]DK | 0.9241 | 1 | 2 [<1%] |
| | #4991 | 2 | TEFGITS[167]DKL | 0.0155 | 1 | |
| **S550** | #4993 | 2 | MMALQTDIVDLQRS[167]PMGR | 0.9977 | 1 | 23 [2%] |
| | #4993 | 3 | MM[147]ALQTDIVDLQRS[167]PM[147]GR | 0.9966 | 6 | |
| | #4993 | 3 | MMALQTDIVDLQRS[167]PM[147]GR | 0.9964 | 4 | |
| | #4993 | 3 | M[147]MALQTDIVDLQRS[167]PM[147]GR | 0.9912 | 4 | |
| | #4993 | 3 | MM[147]ALQTDIVDLQRS[167]PMGR | 0.9891 | 2 | |
| | #4993 | 3 | MMALQTDIVDLQRS[167]PMGR | 0.8634 | 1 | |
| | #4993 | 2 | MMALQTDIVDLQRS[167]PM[147]GR | 0.2819 | 1 | |
| | #4993 | 3 | M[147]M[147]ALQTDIVDLQRS[167]PM[147]GR | 0.1863 | 2 | |
| | #4899 | 3 | MM[147]ALQTDIVDLQRS[167]PM[147]GR | 0.0695 | 1 | |
| | #4993 | 3 | QTDIVDLQRS[167]PM[147]GR_ETD | 0.0262 | 1 | |
| **S600** | #4882 | 2 | LLLQAIQS[167]FEK | 0.9999 | 4 | 18 [2%] |
| | #4993 | 3 | LLLQAIQS[167]FEKK_ETD | 0.9999 | 1 | |
| | #4993 | 2 | LLLQAIQS[167]FEK | 0.9998 | 4 | |
| | #4993 | 2 | LLLQAIQS[167]FEKK | 0.9998 | 2 | |
| | #4899 | 2 | LLLQAIQS[167]FEK | 0.9996 | 4 | |
| | #4898 | 2 | LLLQAIQS[167]FEK | 0.9994 | 3 | |
| **S634** | #4899 | 2 | VEEVVS[167]LM[147]NEDEK | 1 | 9 | 106 [10%] |
| | #4882 | 2 | VEEVVS[167]LM[147]NEDEK | 1 | 6 | |
| | #4898 | 2 | VEEVVS[167]LM[147]NEDEK | 1 | 6 | |
| | #4899 | 3 | VEEVVS[167]LM[147]NEDEK_ETD | 1 | 4 | |
| | #4899 | 2 | VEEVVS[167]LMNEDEK | 1 | 12 | |
| | #4882 | 2 | VEEVVS[167]LMNEDEK | 1 | 7 | |
| | #4898 | 2 | VEEVVS[167]LMNEDEK | 1 | 11 | |
| | #4993 | 2 | VEEVVS[167]LM[147]NEDEK | 0.9999 | 10 | |
| | #4993 | 3 | VEEVVS[167]LM[147]NEDEK_ETD | 0.9999 | 5 | |
| | #4993 | 2 | VEEVVS[167]LMNEDEK | 0.9999 | 13 | |
| | #4993 | 2 | VEEVVS[167]LMNEDEKTVVR | 0.9999 | 2 | |
| | #4993 | 3 | ALELLPKVEEVVS[167]LM[147]NEDEKTVVR | 0.9996 | 7 | |
| | #4993 | 2 | VEEVVS[167]LM[147]NEDEKTVVR | 0.9995 | 2 | |
| | #4882 | 3 | VEEVVS[167]LM[147]NEDEK_ETD | 0.9992 | 2 | |
| | #4993 | 3 | ALELLPKVEEVVS[167]LM[147]NEDEK | 0.9991 | 5 | |
| | #4993 | 3 | VEEVVS[167]LMNEDEKTVVR | 0.9991 | 1 | |
| | #4993 | 3 | ALELLPKVEEVVS[167]LMNEDEK | 0.9834 | 1 | |
| | #4993 | 4 | ALELLPKVEEVVS[167]LM[147]NEDEKTVVR_ETD | 0.8504 | 1 | |
| | #4993 | 3 | VEEVVS[167]LM[147]NEDEKTVVR | 0.0451 | 2 | |
| **S670** | #4993 | 2 | VRGPVS[167]GSPDSMNASR | 0.9963 | 3 | 8 [<1%] |
| | #4993 | 2 | VRGPVS[167]GS[167]PDSMNASR | 0.9877 | 1 | |
| | #5038 | 2 | PVS[167]GSPDSM[147]NASRLSQPGQL | 0.2765 | 1 | |

| | | | | | | |
|---|---|---|---|---|---|---|
| | #5038 | 2 | KVRGPVS[167]GSPDSM[147]NASRL | 0.0246 | 2 | |
| | #4993 | 2 | VRGPVS[167]GSPDSM[147]N | 0.0173 | 1 | |
| **S672** | #4882 | 2 | GPVSGS[167]PDSMNASR | 1 | 4 | |
| | #4898 | 2 | GPVSGS[167]PDSMNASR | 1 | 9 | |
| | #4991 | 2 | PVSGS[167]PDSM[147]NASRLSQPGQL | 1 | 7 | |
| | #4882 | 3 | VRGPVSGS[167]PDSMNASR_ETD | 1 | 1 | |
| | #4993 | 2 | GPVSGS[167]PDSMNASR | 0.9999 | 9 | |
| | #5038 | 2 | KVRGPVSGS[167]PDSM[147]NASRL | 0.9999 | 5 | |
| | #5038 | 3 | KVRGPVSGS[167]PDSM[147]NASRL | 0.9999 | 13 | |
| | #5038 | 3 | KVRGPVSGS[167]PDSM[147]NASRLSQPGQL | 0.9999 | 10 | |
| | #5038 | 4 | KVRGPVSGS[167]PDSM[147]NASRLSQPGQL_ETD | 0.9999 | 2 | |
| | #5038 | 2 | PVSGS[167]PDSM[147]NASRLSQPGQL | 0.9999 | 3 | |
| | #4993 | 3 | VRGPVSGS[167]PDSM[147]NASR_ETD | 0.9999 | 25 | 140 [13%] |
| | #4993 | 2 | VRGPVSGS[167]PDSMNASR | 0.9999 | 11 | |
| | #4993 | 3 | VRGPVSGS[167]PDSMNASR_ETD | 0.9999 | 15 | |
| | #4899 | 2 | GPVSGS[167]PDSMNASR | 0.9997 | 1 | |
| | #4993 | 2 | VRGPVSGS[167]PDSM[147]NASR | 0.9996 | 17 | |
| | #4899 | 3 | VRGPVSGS[167]PDSMNASR_ETD | 0.9995 | 1 | |
| | #5038 | 2 | KVRGPVSGS[167]PDSM[147]NASRLSQPGQL | 0.9767 | 2 | |
| | #4882 | 3 | VRGPVSGS[167]PDSM[147]NASR_ETD | 0.4264 | 1 | |
| | #4882 | 2 | VRGPVSGS[167]PDSMNASR | 0.1267 | 2 | |
| | #4991 | 2 | PVSGS[167]PDSM[147]NASRL | 0.0294 | 1 | |
| | #4993 | 2 | VRGPVSGS[167]PDS[167]MNASR | 0.0112 | 1 | |
| **S675** | #4993 | 2 | VRGPVSGSPDS[167]MNASR | 0.9953 | 1 | |
| | #4993 | 3 | VRGPVSGSPDS[167]M[147]NASR_ETD | 0.1245 | 1 | 3 [<1%] |
| | #4991 | 2 | PVSGSPDS[167]M[147]NASRL | 0.055 | 1 | |
| **S682** | #4991 | 2 | ASRLS[167]QPGQL | 0.7767 | 9 | |
| | #5038 | 2 | ASRLS[167]QPGQL | 0.1888 | 4 | 22 [2%] |
| | #5038 | 2 | SRLS[167]QPGQL | 0.0858 | 5 | |
| | #4991 | 2 | SRLS[167]QPGQL | 0.0269 | 4 | |
| **S695** | #4899 | 3 | LSQPGQLM[147]SQPSTAS[167]NSLPEPAK | 1 | 1 | |
| | #4882 | 3 | LSQPGQLM[147]SQPSTAS[167]NSLPEPAK | 1 | 3 | |
| | #4898 | 3 | LSQPGQLM[147]SQPSTAS[167]NSLPEPAK | 1 | 5 | |
| | #4993 | 2 | LSQPGQLM[147]SQPSTAS[167]NSLPEPAK | 0.9999 | 1 | |
| | #4993 | 3 | LSQPGQLM[147]SQPSTAS[167]NSLPEPAK | 0.9999 | 3 | |
| | #4882 | 2 | LSQPGQLM[147]SQPSTAS[167]NSLPEPAK | 0.9999 | 2 | |
| | #4898 | 3 | LSQPGQLM[147]SQPSTAS[167]NSLPEPAKK | 0.9999 | 1 | 24 [2%] |
| | #4993 | 3 | LSQPGQLMSQPSTAS[167]NSLPEPAK | 0.9999 | 2 | |
| | #4882 | 3 | LSQPGQLMSQPSTAS[167]NSLPEPAK | 0.9999 | 1 | |
| | #4898 | 3 | LSQPGQLMSQPSTAS[167]NSLPEPAK | 0.9999 | 1 | |
| | #4898 | 2 | LSQPGQLM[147]SQPSTAS[167]NSLPEPAK | 0.9989 | 1 | |

| | | | | | | |
|---|---|---|---|---|---|---|
| | #4899 | 3 | LSQPGQLM[147]SQPSTAS[167]NSLPEPAKK | 0.9942 | 1 | |
| | #4898 | 3 | LSQPGQLMSQPSTAS[167]NSLPEPAKK | 0.9911 | 1 | |
| | #4882 | 3 | LSQPGQLM[147]SQPSTAS[167]NSLPEPAKK | 0.3479 | 1 | |
| **S697** | #4899 | 2 | LSQPGQLM[147]SQPSTASNS[167]LPEPAK | 1 | 4 | |
| | #4899 | 3 | LSQPGQLM[147]SQPSTASNS[167]LPEPAK | 1 | 6 | |
| | #4882 | 2 | LSQPGQLM[147]SQPSTASNS[167]LPEPAK | 1 | 2 | |
| | #4882 | 3 | LSQPGQLM[147]SQPSTASNS[167]LPEPAK | 1 | 1 | |
| | #4898 | 2 | LSQPGQLM[147]SQPSTASNS[167]LPEPAK | 1 | 4 | |
| | #4898 | 3 | LSQPGQLM[147]SQPSTASNS[167]LPEPAK | 1 | 3 | |
| | #4899 | 2 | LSQPGQLMSQPSTASNS[167]LPEPAK | 1 | 4 | |
| | #4899 | 3 | LSQPGQLMSQPSTASNS[167]LPEPAK | 1 | 4 | |
| | #4882 | 3 | LSQPGQLMSQPSTASNS[167]LPEPAK | 1 | 3 | |
| | #4993 | 2 | LSQPGQLM[147]SQPSTASNS[167]LPEPAK | 0.9999 | 3 | |
| | #4993 | 2 | LSQPGQLMSQPSTASNS[167]LPEPAK | 0.9999 | 5 | |
| | #4882 | 2 | LSQPGQLMSQPSTASNS[167]LPEPAK | 0.9999 | 1 | |
| | #4898 | 2 | LSQPGQLMSQPSTASNS[167]LPEPAK | 0.9999 | 5 | |
| | #4898 | 3 | LSQPGQLMSQPSTASNS[167]LPEPAK | 0.9999 | 3 | 100 [9%] |
| | #4899 | 3 | LSQPGQLMSQPSTASNS[167]LPEPAKK | 0.9999 | 8 | |
| | #4899 | 3 | LSQPGQLM[147]SQPSTASNS[167]LPEPAKK | 0.9998 | 8 | |
| | #4882 | 3 | LSQPGQLM[147]SQPSTASNS[167]LPEPAKK | 0.9998 | 6 | |
| | #4898 | 3 | LSQPGQLM[147]SQPSTASNS[167]LPEPAKK | 0.9998 | 7 | |
| | #4993 | 3 | LSQPGQLM[147]SQPSTASNS[167]LPEPAK | 0.9997 | 1 | |
| | #4993 | 3 | LSQPGQLMSQPSTASNS[167]LPEPAK | 0.9997 | 2 | |
| | #4898 | 3 | LSQPGQLMSQPSTASNS[167]LPEPAKK | 0.9995 | 4 | |
| | #4993 | 3 | LSQPGQLM[147]SQPSTASNS[167]LPEPAKK | 0.9993 | 5 | |
| | #4993 | 2 | LSQPGQLMSQPSTASNS[167]LPEPAKK | 0.9992 | 1 | |
| | #4882 | 3 | LSQPGQLMSQPSTASNS[167]LPEPAKK | 0.9984 | 4 | |
| | #4898 | 2 | LSQPGQLM[147]SQPSTASNS[167]LPEPAKK | 0.9925 | 1 | |
| | #4993 | 3 | LSQPGQLMSQPSTASNS[167]LPEPAKK | 0.3764 | 4 | |
| | #4993 | 3 | LSQPGQLM[147]SQPST[181]ASNS[167]LPEPAKK | 0.1228 | 1 | |
| **S733** | #4991 | 2 | AIQDTVREQDQS[167]F | 1 | 6 | |
| | #4991 | 2 | AIQDTVREQDQS[167]FTAL | 1 | 4 | |
| | #4991 | 2 | ENAIQDTVREQDQS[167]F | 1 | 5 | |
| | #4991 | 2 | IQDTVREQDQS[167]FTAL | 1 | 4 | |
| | #4991 | 2 | NAIQDTVREQDQS[167]F | 1 | 8 | |
| | #4991 | 2 | NAIQDTVREQDQS[167]FTA | 1 | 6 | |
| | #5038 | 2 | AIQDTVREQDQS[167]FTAL | 0.9999 | 4 | |
| | #5038 | 3 | DTVREQDQS[167]FTAL_ETD | 0.9999 | 1 | |
| | #5038 | 2 | ENAIQDTVREQDQS[167]F | 0.9999 | 5 | |
| | #5038 | 3 | ENAIQDTVREQDQS[167]F_ETD | 0.9999 | 3 | |
| | #4898 | 3 | EQDQS[167]FTALDWSWLQTEEEEHSCLEQAS | 0.9999 | 1 | |
| | #5038 | 2 | IQDTVREQDQS[167]F | 0.9999 | 5 | |
| | #5038 | 3 | IQDTVREQDQS[167]F_ETD | 0.9999 | 2 | |

| | | | | | | |
|---|---|---|---|---|---|---|
| | #5038 | 2 | IQDTVREQDQS[167]FTAL | 0.9999 | 4 | |
| | #5038 | 3 | IQDTVREQDQS[167]FTAL_ETD | 0.9999 | 2 | |
| | #5038 | 2 | NAIQDTVREQDQS[167]F | 0.9999 | 7 | |
| | #5038 | 3 | NAIQDTVREQDQS[167]F_ETD | 0.9999 | 2 | 144 [13%] |
| | #5038 | 2 | NAIQDTVREQDQS[167]FTA | 0.9999 | 3 | |
| | #5038 | 2 | NAIQDTVREQDQS[167]FTAL | 0.9999 | 4 | |
| | #5038 | 3 | TVREQDQS[167]FTAL_ETD | 0.9999 | 2 | |
| | #4991 | 2 | IQDTVREQDQS[167]F | 0.9996 | 9 | |
| | #4991 | 2 | DTVREQDQS[167]FTAL | 0.999 | 4 | |
| | #5038 | 2 | TVREQDQS[167]FTAL | 0.9986 | 19 | |
| | #5038 | 2 | AIQDTVREQDQS[167]F | 0.9982 | 3 | |
| | #4991 | 2 | NAIQDTVREQDQS[167]FTAL | 0.998 | 4 | |
| | #5038 | 2 | VREQDQS[167]FTAL | 0.9958 | 3 | |
| | #4991 | 2 | TVREQDQS[167]FTAL | 0.9946 | 9 | |
| | #5038 | 2 | DTVREQDQS[167]FTAL | 0.9927 | 7 | |
| | #5038 | 3 | NAIQDTVREQDQS[167]FTA_ETD | 0.9905 | 2 | |
| | #4991 | 2 | VREQDQS[167]FTAL | 0.9876 | 2 | |
| | #5038 | 3 | NAIQDTVREQDQS[167]FTAL | 0.2221 | 2 | |
| | #4882 | 2 | EQDQS[167]FTALDWS | 0.1205 | 1 | |
| | #4993 | 2 | EQDQS[167]FTALD | 0.0175 | 1 | |
| **S740** | #5038 | 2 | TALDWS[167]WLQT | 0.0143 | 1 | 1 [<1%] |

**Notes:**

**T[181]** indicates pThr residue in peptide.
**S[167]** indicates pSer residue in peptide.
**M[147]** indicates peptide containing oxidized Met residue.
**_ETD** indicates peptide identification via an Electron-Transfer Dissociation (ETD) MS/MS spectrum.
Peptides with nsp probability < 0.01 discarded.
