# Supplementary material for: Tyrosine Phosphorylation Allows Integration of Multiple Signaling Inputs by IKKβ
Source: PLoS One. 2013 Dec 27;8(12):e84497. doi: 10.1371/journal.pone.0084497 (PMC3873999; doi:10.1371/journal.pone.0084497)
Supplement: Table S4 — Peptide Analysis of IKKβ: Total Peptides. (PDF) [file pone.0084497.s006.pdf]

Table S4: Peptide Analysis of IKKβ: Total Peptides

(see Notes at end)

Prep #4882	Prep #4898	Prep #4899	Prep #4991	Prep #4993	Prep #5038	Starting Residue #	Ending Residue #	Phospho AA	Phospho AA Residue	Precursor Ion Charge	Peptide Sequence	NSP adjusted probability	Number Sibling Peptides	Spectral Count
				x		4	18	S	4	2	S[167]PSLTTQTCGAWEM[147]K	0.9947	7	1
	x					4	18	S	4	2	S[167]PSLTTQTCGAWEM[147]K	0.9933	7	2
				x		4	18	S	6	2	S[167]PSLTTQTCGAWEMK	0.9892	7	1
				x		4	18	S	6	2	SPS[167]LTTQTCGAWEM[147]K	0.9857	7	2
	x					4	18	S	6	2	SPS[167]LTTQTCGAWEM[147]K	0.0355	7	1
x						21	31	T	23	2	LGT[181]GGFGNVIR	1	7	4
		x				21	31	T	23	2	LGT[181]GGFGNVIR	0.9999	7	4
				x		21	31	T	23	2	LGT[181]GGFGNVIR	0.9999	7	4
	x					21	31	T	23	2	LGT[181]GGFGNVIR	0.9998	7	5
		x				164	171	Y	169	2	IIDLGY[243]AK	0.9993	7	3
				x		164	171	Y	169	2	IIDLGY[243]AK	0.999	7	9
x						164	171	Y	169	2	IIDLGY[243]AK	0.9969	7	2
			x			168	178	Y	169	2	GY[243]AKELDQGSL	0.998	7	4
	x					172	186	T	180	2	ELDQGSLCT[181]SFVGTL	0.0098	7	1
				x		172	187	T	180	2	ELDQGSLCT[181]SFVGTLQ	0.0193	7	1
	x					172	187	T	180	2	ELDQGSLCT[181]SFVGTLQ	0.0078	7	1
x						172	188	T	180	2	ELDQGSLCT[181]SFVGTLQY	0.9977	7	3
				x		172	188	T	180	2	ELDQGSLCT[181]SFVGTLQY	0.9921	7	2
		x				172	188	T	180	2	ELDQGSLCT[181]SFVGTLQY	0.0508	7	1
	x					172	198	T	180	3	ELDQGSLCT[181]SFVGTLQYLAPELLEQQK	0.9999	7	2
				x		172	198	T	180	3	ELDQGSLCT[181]SFVGTLQYLAPELLEQQK	0.9997	7	1
				x		172	184	S	181	2	ELDQGSLCTS[167]FVG	0.0034	7	1
				x		172	187	S	181	2	ELDQGSLCTS[167]FVGTLQ	0.0587	7	1
	x					172	187	S	181	2	ELDQGSLCTS[167]FVGTLQ	0.0287	7	2
	x					172	188	S	181	2	ELDQGSLCTS[167]FVGTLQY	0.9943	7	4
				x		172	188	S	181	2	ELDQGSLCTS[167]FVGTLQY	0.9898	7	2
		x				172	188	S	181	2	ELDQGSLCTS[167]FVGTLQY	0.819	7	2
			x			194	205	Y	199	2	LEQQKY[243]TVTVDY	0.0167	7	1
				x		237	254	S	239	3	QKS[167]EVDIVVSEDLNGTVK	0.1177	7	1
	x					237	254	S	239	3	QKS[167]EVDIVVSEDLNGTVK	0.1064	7	1
x						237	254	S	239	3	QKS[167]EVDIVVSEDLNGTVK	0.0896	7	3
x						239	254	S	246	2	SEVDIVVS[167]EDLNGTVK	1	7	4
	x					239	254	S	246	2	SEVDIVVS[167]EDLNGTVK	1	7	4
		x				239	254	S	246	2	SEVDIVVS[167]EDLNGTVK	0.9996	7	2
x						239	254	S	246	3	SEVDIVVS[167]EDLNGTVK_ETD	1	7	3
	x					239	254	S	246	3	SEVDIVVS[167]EDLNGTVK_ETD	0.9999	7	1
					x	243	255	S	246	2	IVVS[167]EDLNGTVKF	0.9999	7	5
			x			243	255	S	246	2	IVVS[167]EDLNGTVKF	0.9996	7	2
					x	252	265	S	256	2	TVKFS[167]SSLPYPNNL	0.9747	7	1
				x		255	272	S	256	2	FS[167]SSLPYPNNLNSVLAER	0.9999	7	2
		x				255	272	S	256	2	FS[167]SSLPYPNNLNSVLAER	0.9992	7	1
	x					255	272	S	256	2	FS[167]SSLPYPNNLNSVLAER	0.9986	7	1
					x	252	265	S	257	2	TVKFSS[167]SLPYPNNL	0.9948	7	5
					x	253	265	S	257	2	VKFSS[167]SLPYPNNL	0.0203	7	1
		x				255	272	S	257	2	FSS[167]SLPYPNNLNSVLAER	1	7	2
x						255	272	S	257	2	FSS[167]SLPYPNNLNSVLAER	0.9999	7	1
				x		255	272	S	257	2	FSS[167]SLPYPNNLNSVLAER	0.9999	7	1
	x					255	272	S	257	2	FSS[167]SLPYPNNLNSVLAER	0.9998	7	1
	x					255	272	S	257	3	FSS[167]SLPYPNNLNSVLAER	0.3826	7	1
					x	252	265	S	258	2	TVKFSSS[167]LPYPNNL	0.9942	7	5
			x			252	265	S	258	2	TVKFSSS[167]LPYPNNL	0.0396	7	1
					x	253	265	S	258	2	VKFSSS[167]LPYPNNL	0.0598	7	1
x						255	272	S	258	2	FSSS[167]LPYPNNLNSVLAER	1	7	3
		x				255	272	S	258	2	FSSS[167]LPYPNNLNSVLAER	1	7	1
	x					255	272	S	258	2	FSSS[167]LPYPNNLNSVLAER	0.9999	7	2
				x		255	272	S	258	2	FSSS[167]LPYPNNLNSVLAER	0.9999	7	1
	x					255	272	S	258	3	FSSS[167]LPYPNNLNSVLAER	0.9998	7	1
x						255	272	S	258	3	FSSS[167]LPYPNNLNSVLAER	0.7885	7	1

		x				255	272	Y	261	2	FSSSLPY[243]PNNLNSVLAER	0.9999	7	3
x						255	272	Y	261	3	FSSSLPY[243]PNNLNSVLAER	0.1521	7	1
					x	256	265	Y	261	2	SSSLPY[243]PNNL	0.1344	7	3
x						255	272	S	267	2	FSSSLPYPNNLNS[167]VLAER	1	7	6
		x				255	272	S	267	2	FSSSLPYPNNLNS[167]VLAER	1	7	4
		x				255	272	S	267	3	FSSSLPYPNNLNS[167]VLAER	1	7	4
				x		255	272	S	267	2	FSSSLPYPNNLNS[167]VLAER	0.9999	7	4
				x		255	272	S	267	3	FSSSLPYPNNLNS[167]VLAER	0.9998	7	1
x						255	272	S	267	3	FSSSLPYPNNLNS[167]VLAER	0.9987	7	1
					x	256	269	S	267	2	SSSLPYPNNLNS[167]VL	0.9922	7	4
			x			256	269	S	267	2	SSSLPYPNNLNS[167]VL	0.9891	7	4
				x		262	272	S	267	2	PNNLNS[167]VLAER	0.1369	7	1
x						264	272	S	267	2	NLNS[167]VLAER	0.992	7	4
		x				264	272	S	267	2	NLNS[167]VLAER	0.9895	7	2
	x					264	272	S	267	2	NLNS[167]VLAER	0.8969	7	3
				x		264	272	S	267	2	NLNS[167]VLAER	0.3159	7	3
					x	318	329	T	324	2	VTGTIHT[181]YPVTE	0.0361	7	1
			x			317	333	Y	325	2	M[147]VTGTIHTY[243]PVTEDESL	0.2753	7	2
					x	318	333	Y	325	2	VTGTIHTY[243]PVTEDESL	0.3137	7	3
			x			318	333	Y	325	2	VTGTIHTY[243]PVTEDESL	0.1539	7	2
	x					311	337	S	332	3	LVHILNM[147]VTGTIHTYPVTEDES[167]LQSLK	1	7	2
				x		311	337	S	332	3	LVHILNM[147]VTGTIHTYPVTEDES[167]LQSLK	0.9999	7	2
	x					311	337	S	332	4	LVHILNM[147]VTGTIHTYPVTEDES[167]LQSLK_ETD	0.9995	7	1
				x		311	337	S	332	3	LVHILNMVTGTIHTYPVTEDES[167]LQSLK	0.9999	7	2
	x					311	337	S	332	3	LVHILNMVTGTIHTYPVTEDES[167]LQSLK	0.9985	7	1
	x					314	337	S	332	3	ILNM[147]VTGTIHTYPVTEDES[167]LQSLK	0.9976	7	1
x						314	337	S	332	3	ILNM[147]VTGTIHTYPVTEDES[167]LQSLK	0.0116	7	1
					x	316	333	S	332	2	NM[147]VTGTIHTYPVTEDES[167]L	0.9999	7	1
					x	317	333	S	332	2	M[147]VTGTIHTYPVTEDES[167]L	0.7892	7	3
			x			317	333	S	332	2	M[147]VTGTIHTYPVTEDES[167]L	0.2757	7	1
					x	317	333	S	332	2	MVTGTIHTYPVTEDES[167]L	0.0849	7	1
x						317	337	S	332	3	M[147]VTGTIHTYPVTEDES[167]LQSLK	1	7	3
	x					317	337	S	332	3	M[147]VTGTIHTYPVTEDES[167]LQSLK	1	7	3
		x				317	337	S	332	2	M[147]VTGTIHTYPVTEDES[167]LQSLK	0.1117	7	1
				x		317	337	S	332	3	M[147]VTGTIHTYPVTEDES[167]LQSLK	0.0701	7	1
				x		317	337	S	332	2	M[147]VTGTIHTYPVTEDES[167]LQSLK	0.055	7	1
				x		317	337	S	332	2	MVTGTIHTYPVTEDES[167]LQSLK	0.9999	7	3
x						317	337	S	332	3	MVTGTIHTYPVTEDES[167]LQSLK	0.9988	7	4
				x		317	337	S	332	3	MVTGTIHTYPVTEDES[167]LQSLK	0.9951	7	4
		x				317	337	S	332	3	MVTGTIHTYPVTEDES[167]LQSLK	0.4076	7	3
	x					317	337	S	332	3	MVTGTIHTYPVTEDES[167]LQSLK	0.1384	7	3
			x			318	333	S	332	2	VTGTIHTYPVTEDES[167]L	1	7	6
					x	318	333	S	332	2	VTGTIHTYPVTEDES[167]L	0.9999	7	4
				x		320	337	S	332	3	GTIHTYPVTEDES[167]LQSLK	0.0075	7	1
				x		326	337	S	332	2	PVTEDES[167]LQSLK	0.995	7	4
	x					311	337	S	335	3	LVHILNM[147]VTGTIHTYPVTEDESLQS[167]LK	1	7	3
		x				311	337	S	335	3	LVHILNM[147]VTGTIHTYPVTEDESLQS[167]LK	1	7	1
				x		311	337	S	335	3	LVHILNM[147]VTGTIHTYPVTEDESLQS[167]LK	0.9915	7	6
	x					311	337	S	335	4	LVHILNM[147]VTGTIHTYPVTEDESLQS[167]LK_ETD	1	7	1
				x		311	337	S	335	4	LVHILNM[147]VTGTIHTYPVTEDESLQS[167]LK_ETD	0.9998	7	6
	x					311	337	S	335	3	LVHILNMVTGTIHTYPVTEDESLQS[167]LK	1	7	4
		x				311	337	S	335	3	LVHILNMVTGTIHTYPVTEDESLQS[167]LK	0.9999	7	1
				x		311	337	S	335	3	LVHILNMVTGTIHTYPVTEDESLQS[167]LK	0.9999	7	9
				x		311	337	S	335	4	LVHILNMVTGTIHTYPVTEDESLQS[167]LK_ETD	0.9998	7	8
	x					311	337	S	335	4	LVHILNMVTGTIHTYPVTEDESLQS[167]LK_ETD	0.9996	7	2
				x		311	339	S	335	4	LVHILNM[147]VTGTIHTYPVTEDESLQS[167]LKAR_ETD	0.9979	7	1
	x					314	337	S	335	3	ILNM[147]VTGTIHTYPVTEDESLQS[167]LK	1	7	4
		x				314	337	S	335	3	ILNM[147]VTGTIHTYPVTEDESLQS[167]LK	0.995	7	3
				x		314	337	S	335	3	ILNM[147]VTGTIHTYPVTEDESLQS[167]LK	0.9897	7	2
x						314	337	S	335	3	ILNM[147]VTGTIHTYPVTEDESLQS[167]LK	0.0356	7	2
	x					314	337	S	335	3	ILNMVTGTIHTYPVTEDESLQS[167]LK	1	7	2
				x		314	337	S	335	3	ILNMVTGTIHTYPVTEDESLQS[167]LK	0.0768	7	1
		x				314	337	S	335	3	ILNMVTGTIHTYPVTEDESLQS[167]LK	0.0449	7	1
				x		316	337	S	335	3	NM[147]VTGTIHTYPVTEDESLQS[167]LK	0.9157	7	1
		x				317	337	S	335	3	M[147]VTGTIHTYPVTEDESLQS[167]LK	1	7	11
x						317	337	S	335	2	M[147]VTGTIHTYPVTEDESLQS[167]LK	0.9994	7	3
	x					317	337	S	335	3	M[147]VTGTIHTYPVTEDESLQS[167]LK	0.9973	7	7
				x		317	337	S	335	3	M[147]VTGTIHTYPVTEDESLQS[167]LK	0.944	7	5

	x					317	337	S	335	2	M[147]VTGTIHTYPVTEDESLQS[167]LK	0.182	7	1
		x				317	337	S	335	2	M[147]VTGTIHTYPVTEDESLQS[167]LK	0.1418	7	1
				x		317	337	S	335	2	M[147]VTGTIHTYPVTEDESLQS[167]LK	0.0507	7	1
x						317	337	S	335	3	M[147]VTGTIHTYPVTEDESLQS[167]LK	0.05	7	2
	x					317	337	S	335	2	MVTGTIHTYPVTEDESLQS[167]LK	1	7	6
	x					317	337	S	335	3	MVTGTIHTYPVTEDESLQS[167]LK	1	7	7
		x				317	337	S	335	3	MVTGTIHTYPVTEDESLQS[167]LK	1	7	11
				x		317	337	S	335	2	MVTGTIHTYPVTEDESLQS[167]LK	0.9999	7	6
x						317	337	S	335	2	MVTGTIHTYPVTEDESLQS[167]LK	0.9997	7	3
x						317	337	S	335	3	MVTGTIHTYPVTEDESLQS[167]LK	0.9993	7	2
				x		317	337	S	335	3	MVTGTIHTYPVTEDESLQS[167]LK	0.9989	7	8
		x				317	337	S	335	2	MVTGTIHTYPVTEDESLQS[167]LK	0.9972	7	2
				x		320	337	S	335	2	GTIHTYPVTEDESLQS[167]LK	0.9961	7	2
				x		320	337	S	335	3	GTIHTYPVTEDESLQS[167]LK	0.0088	7	1
				x		324	337	S	335	2	TYPVTEDESLQS[167]LK	0.9967	7	1
				x		326	337	S	335	2	PVTEDESLQS[167]LK	0.9999	7	5
		x				326	337	S	335	2	PVTEDESLQS[167]LK	0.9954	7	3
x						326	337	S	335	2	PVTEDESLQS[167]LK	0.9931	7	4
	x					326	337	S	335	2	PVTEDESLQS[167]LK	0.9915	7	1
				x		326	339	S	335	3	PVTEDESLQS[167]LKAR_ETD	0.9999	7	3
				x		340	352	T	344	2	IQQDT[181]GIPEEDQE	0.0004	7	1
	x					340	375	T	368	4	IQQDTGIPEEDQELLQEAGLALIPDKPAT[181]QCISDGK	0.0012	7	1
			x			389	398	S	393	2	LFDNS[167]KITYE	0.1022	7	2
		x				395	412	T	396	3	IT[181]YETQISPRPQPESVSC	0.0082	7	1
x						395	404	Y	397	2	ITY[243]ETQISPR	0.9893	7	4
		x				395	404	Y	397	2	ITY[243]ETQISPR	0.9874	7	1
				x		395	404	Y	397	2	ITY[243]ETQISPR	0.0455	7	3
	x					395	404	Y	397	2	ITY[243]ETQISPR	0.0273	7	1
		x				395	418	Y	397	3	ITY[243]ETQISPRPQPESVSCILQEPK	0.9999	7	1
		x				395	404	T	399	2	ITYET[181]QISPR	0.1275	7	1
				x		395	404	T	399	2	ITYET[181]QISPR	0.0309	7	1
x						395	404	S	402	2	ITYETQIS[167]PR	0.9997	7	12
		x				395	404	S	402	2	ITYETQIS[167]PR	0.999	7	13
	x					395	404	S	402	2	ITYETQIS[167]PR	0.9982	7	15
				x		395	404	S	402	2	ITYETQIS[167]PR	0.9929	7	8
				x		395	411	S	402	2	ITYETQIS[167]PRPQPESVS	0.9861	7	4
	x					395	412	S	402	2	ITYETQIS[167]PRPQPESVSC	1	7	4
				x		395	412	S	402	2	ITYETQIS[167]PRPQPESVSC	0.9999	7	4
		x				395	412	S	402	2	ITYETQIS[167]PRPQPESVSC	0.9984	7	4
	x					395	412	S	402	3	ITYETQIS[167]PRPQPESVSC	0.2247	7	2
				x		395	412	S	402	3	ITYETQIS[167]PRPQPESVSC	0.1294	7	3
		x				395	412	S	402	3	ITYETQIS[167]PRPQPESVSC	0.0296	7	1
x						395	412	S	402	3	ITYETQIS[167]PRPQPESVSC	0.0071	7	1
x						395	418	S	402	3	ITYETQIS[167]PRPQPESVSCILQEPK	1	7	3
	x					395	418	S	402	3	ITYETQIS[167]PRPQPESVSCILQEPK	1	7	4
		x				395	418	S	402	3	ITYETQIS[167]PRPQPESVSCILQEPK	1	7	7
				x		395	418	S	402	3	ITYETQIS[167]PRPQPESVSCILQEPK	0.9999	7	5
	x					395	418	S	402	4	ITYETQIS[167]PRPQPESVSCILQEPK_ETD	0.9997	7	1
				x		395	418	S	402	4	ITYETQIS[167]PRPQPESVSCILQEPK_ETD	0.9831	7	1
			x			399	410	S	402	2	TQIS[167]PRPQPESV	0.9985	7	8
					x	399	410	S	402	2	TQIS[167]PRPQPESV	0.9943	7	10
			x			399	411	S	402	2	TQIS[167]PRPQPESVS	0.9884	7	8
					x	399	411	S	402	2	TQIS[167]PRPQPESVS	0.9713	7	8
		x				395	418	S	409	3	ITYETQISPRPQPES[167]VSCILQEPK	0.9953	7	1
	x					395	418	S	409	3	ITYETQISPRPQPES[167]VSCILQEPK	0.9827	7	1
x						405	418	S	409	2	PQPES[167]VSCILQEPK	0.9987	7	4
	x					405	418	S	409	2	PQPES[167]VSCILQEPK	0.9973	7	4
		x				405	418	S	409	2	PQPES[167]VSCILQEPK	0.9964	7	4
				x		405	418	S	409	2	PQPES[167]VSCILQEPK	0.9951	7	4
		x				405	418	S	411	2	PQPESVS[167]CILQEPK	0.9928	7	1
				x		405	418	S	411	2	PQPESVS[167]CILQEPK	0.9881	7	1
				x		470	480	S	474	2	NSMAS[167]M[147]SQQLK	0.9797	7	1
				x		470	480	S	474	2	NSMAS[167]MSQQLK	0.9998	7	4
	x					470	480	S	474	2	NSMAS[167]MSQQLK	0.8299	7	1
				x		470	480	S	476	2	NSMASM[147]S[167]QQLK	0.9911	7	1
				x		470	480	S	476	2	NSMASMS[167]QQLK	0.4202	7	1
					x	486	494	T	498	2	FKT[181]SIQIDL	0.1484	7	2
			x			486	494	T	498	2	FKT[181]SIQIDL	0.052	7	1

					x	486	494	S	499	2	FKTS[167]IQIDL	0.0961	7	2
x						497	509	S	507	2	YSEQTEFGITS[167]DK	0.9241	7	1
			x			501	510	S	507	2	TEFGITS[167]DKL	0.0155	7	1
				x		537	554	S	550	3	M[147]M[147]ALQTDIVDLQRS[167]PM[147]GR	0.1863	7	2
				x		537	554	S	550	3	M[147]MALQTDIVDLQRS[167]PM[147]GR	0.9912	7	4
				x		537	554	S	550	3	MM[147]ALQTDIVDLQRS[167]PM[147]GR	0.9966	7	6
		x				537	554	S	550	3	MM[147]ALQTDIVDLQRS[167]PM[147]GR	0.0695	7	1
				x		537	554	S	550	3	MM[147]ALQTDIVDLQRS[167]PMGR	0.9891	7	2
				x		537	554	S	550	3	MMALQTDIVDLQRS[167]PM[147]GR	0.9964	7	4
				x		537	554	S	550	2	MMALQTDIVDLQRS[167]PM[147]GR	0.2819	7	1
				x		537	554	S	550	2	MMALQTDIVDLQRS[167]PMGR	0.9977	7	1
				x		537	554	S	550	3	MMALQTDIVDLQRS[167]PMGR	0.8634	7	1
				x		541	554	S	550	3	QTDIVDLQRS[167]PM[147]GR_ETD	0.0262	7	1
x						555	568	T	559	2	KQGGT[181]LDDLEEQAR	1	7	3
	x					555	568	T	559	2	KQGGT[181]LDDLEEQAR	0.9999	7	5
		x				555	568	T	559	2	KQGGT[181]LDDLEEQAR	0.9999	7	5
				x		555	568	T	559	2	KQGGT[181]LDDLEEQAR	0.9999	7	9
x						555	568	T	559	3	KQGGT[181]LDDLEEQAR_ETD	1	7	2
	x					555	568	T	559	3	KQGGT[181]LDDLEEQAR_ETD	1	7	6
		x				555	568	T	559	3	KQGGT[181]LDDLEEQAR_ETD	1	7	8
				x		555	568	T	559	3	KQGGT[181]LDDLEEQAR_ETD	0.9999	7	7
x						556	568	T	559	2	QGGT[181]LDDLEEQAR	1	7	4
	x					556	568	T	559	2	QGGT[181]LDDLEEQAR	0.9999	7	5
				x		556	568	T	559	2	QGGT[181]LDDLEEQAR	0.9999	7	4
		x				556	568	T	559	2	QGGT[181]LDDLEEQAR	0.9998	7	4
x						593	603	S	600	2	LLLQAIQS[167]FEK	0.9999	7	4
				x		593	603	S	600	2	LLLQAIQS[167]FEK	0.9998	7	4
		x				593	603	S	600	2	LLLQAIQS[167]FEK	0.9996	7	4
	x					593	603	S	600	2	LLLQAIQS[167]FEK	0.9994	7	3
				x		593	604	S	600	2	LLLQAIQS[167]FEKK	0.9998	7	2
				x		593	604	S	600	3	LLLQAIQS[167]FEKK_ETD	0.9999	7	1
				x		607	614	Y	609	2	VIY[243]TQLSK	0.9963	7	2
x						607	614	Y	609	2	VIY[243]TQLSK	0.991	7	1
x						607	614	T	610	2	VIYT[181]QLSK	0.9907	7	1
				x		607	614	T	610	2	VIYT[181]QLSK	0.976	7	1
				x		622	641	S	634	3	ALELLPKVEEVVS[167]LM[147]NEDEK	0.9991	7	5
				x		622	641	S	634	3	ALELLPKVEEVVS[167]LMNEDEK	0.9834	7	1
				x		622	645	S	634	3	ALELLPKVEEVVS[167]LM[147]NEDEKTVVR	0.9996	7	7
				x		622	645	S	634	4	ALELLPKVEEVVS[167]LM[147]NEDEKTVVR_ETD	0.8504	7	1
x						629	641	S	634	2	VEEVVS[167]LM[147]NEDEK	1	7	6
	x					629	641	S	634	2	VEEVVS[167]LM[147]NEDEK	1	7	6
		x				629	641	S	634	2	VEEVVS[167]LM[147]NEDEK	1	7	9
				x		629	641	S	634	2	VEEVVS[167]LM[147]NEDEK	0.9999	7	10
		x				629	641	S	634	3	VEEVVS[167]LM[147]NEDEK_ETD	1	7	4
				x		629	641	S	634	3	VEEVVS[167]LM[147]NEDEK_ETD	0.9999	7	5
x						629	641	S	634	3	VEEVVS[167]LM[147]NEDEK_ETD	0.9992	7	2
x						629	641	S	634	2	VEEVVS[167]LMNEDEK	1	7	7
	x					629	641	S	634	2	VEEVVS[167]LMNEDEK	1	7	11
		x				629	641	S	634	2	VEEVVS[167]LMNEDEK	1	7	12
				x		629	641	S	634	2	VEEVVS[167]LMNEDEK	0.9999	7	13
				x		629	645	S	634	2	VEEVVS[167]LM[147]NEDEKTVVR	0.9995	7	2
				x		629	645	S	634	3	VEEVVS[167]LM[147]NEDEKTVVR	0.0451	7	2
				x		629	645	S	634	2	VEEVVS[167]LMNEDEKTVVR	0.9999	7	2
				x		629	645	S	634	3	VEEVVS[167]LMNEDEKTVVR	0.9991	7	1
					x	663	681	S	670	2	SKVRGPVS[167]GSPDSM[147]NASRL	0.0026	7	1
					x	663	681	S	670	3	SKVRGPVS[167]GSPDSM[147]NASRL	0.0002	7	1
					x	664	681	S	670	2	KVRGPVS[167]GSPDSM[147]NASRL	0.0246	7	2
				x		665	677	S	670	2	VRGPVS[167]GSPDSM[147]N	0.0173	7	1
				x		665	680	S	670	2	VRGPVS[167]GS[167]PDSMNASR	0.9877	7	1
				x		665	680	S	670	2	VRGPVS[167]GSPDSMNASR	0.9963	7	3
					x	668	687	S	670	2	PVS[167]GSPDSM[147]NASRLSQPGQL	0.2765	7	1
					x	663	681	S	672	3	SKVRGPVSGS[167]PDSM[147]NASRL	0.0018	7	1
					x	664	681	S	672	2	KVRGPVSGS[167]PDSM[147]NASRL	0.9999	7	5
					x	664	681	S	672	3	KVRGPVSGS[167]PDSM[147]NASRL	0.9999	7	13
			x			664	681	S	672	2	KVRGPVSGS[167]PDSM[147]NASRL	0.0003	7	3
					x	664	687	S	672	3	KVRGPVSGS[167]PDSM[147]NASRLSQPGQL	0.9999	7	10
					x	664	687	S	672	2	KVRGPVSGS[167]PDSM[147]NASRLSQPGQL	0.9767	7	2
					x	664	687	S	672	4	KVRGPVSGS[167]PDSM[147]NASRLSQPGQL_ETD	0.9999	7	2

				x		665	678	S	672	2	VRGPVSGS[167]PDSMNA	0.001	7	1
				x		665	680	S	672	2	VRGPVSGS[167]PDSM[147]NASR	0.9996	7	17
				x		665	680	S	672	3	VRGPVSGS[167]PDSM[147]NASR_ETD	0.9999	7	25
x						665	680	S	672	3	VRGPVSGS[167]PDSM[147]NASR_ETD	0.4264	7	1
				x		665	680	S	672	2	VRGPVSGS[167]PDSMNASR	0.9999	7	11
x						665	680	S	672	2	VRGPVSGS[167]PDSMNASR	0.1267	7	2
x						665	680	S	672	3	VRGPVSGS[167]PDSMNASR_ETD	1	7	1
				x		665	680	S	672	3	VRGPVSGS[167]PDSMNASR_ETD	0.9999	7	15
		x				665	680	S	672	3	VRGPVSGS[167]PDSMNASR_ETD	0.9995	7	1
x						667	680	S	672	2	GPVSGS[167]PDSMNASR	1	7	4
	x					667	680	S	672	2	GPVSGS[167]PDSMNASR	1	7	9
				x		667	680	S	672	2	GPVSGS[167]PDSMNASR	0.9999	7	9
		x				667	680	S	672	2	GPVSGS[167]PDSMNASR	0.9997	7	1
			x			668	681	S	672	2	PVSGS[167]PDSM[147]NASRL	0.0294	7	1
			x			668	687	S	672	2	PVSGS[167]PDSM[147]NASRLSQPGQL	1	7	7
					x	668	687	S	672	2	PVSGS[167]PDSM[147]NASRLSQPGQL	0.9999	7	3
				x		665	680	S	675	3	VRGPVSGSPDS[167]M[147]NASR_ETD	0.1245	7	1
				x		665	680	S	675	2	VRGPVSGSPDS[167]MNASR	0.9953	7	1
			x			668	681	S	675	2	PVSGSPDS[167]M[147]NASRL	0.055	7	1
					x	664	687	S	679	3	KVRGPVSGSPDSM[147]NAS[167]RLSQPGQL	0.0006	7	1
					x	664	687	S	682	4	KVRGPVSGSPDSM[147]NASRLS[167]QPGQL_ETD	0.001	7	1
			x			668	687	S	682	2	PVSGSPDSM[147]NASRLS[167]QPGQL	0.0012	7	1
			x			678	687	S	682	2	ASRLS[167]QPGQL	0.7767	7	9
					x	678	687	S	682	2	ASRLS[167]QPGQL	0.1888	7	4
					x	679	687	S	682	2	SRLS[167]QPGQL	0.0858	7	5
			x			679	687	S	682	2	SRLS[167]QPGQL	0.0269	7	4
		x				681	704	T	693	3	LSQPGQLMSQPST[181]ASNSLPEPAKK	0.9948	7	1
x						681	703	S	695	3	LSQPGQLM[147]SQPSTAS[167]NSLPEPAK	1	7	3
	x					681	703	S	695	3	LSQPGQLM[147]SQPSTAS[167]NSLPEPAK	1	7	5
		x				681	703	S	695	3	LSQPGQLM[147]SQPSTAS[167]NSLPEPAK	1	7	1
x						681	703	S	695	2	LSQPGQLM[147]SQPSTAS[167]NSLPEPAK	0.9999	7	2
				x		681	703	S	695	2	LSQPGQLM[147]SQPSTAS[167]NSLPEPAK	0.9999	7	1
				x		681	703	S	695	3	LSQPGQLM[147]SQPSTAS[167]NSLPEPAK	0.9999	7	3
	x					681	703	S	695	2	LSQPGQLM[147]SQPSTAS[167]NSLPEPAK	0.9989	7	1
x						681	703	S	695	3	LSQPGQLMSQPSTAS[167]NSLPEPAK	0.9999	7	1
	x					681	703	S	695	3	LSQPGQLMSQPSTAS[167]NSLPEPAK	0.9999	7	1
				x		681	703	S	695	3	LSQPGQLMSQPSTAS[167]NSLPEPAK	0.9999	7	2
	x					681	704	S	695	3	LSQPGQLM[147]SQPSTAS[167]NSLPEPAKK	0.9999	7	1
		x				681	704	S	695	3	LSQPGQLM[147]SQPSTAS[167]NSLPEPAKK	0.9942	7	1
x						681	704	S	695	3	LSQPGQLM[147]SQPSTAS[167]NSLPEPAKK	0.3479	7	1
	x					681	704	S	695	3	LSQPGQLMSQPSTAS[167]NSLPEPAKK	0.9911	7	1
x						681	703	S	697	2	LSQPGQLM[147]SQPSTASNS[167]LPEPAK	1	7	2
x						681	703	S	697	3	LSQPGQLM[147]SQPSTASNS[167]LPEPAK	1	7	1
	x					681	703	S	697	2	LSQPGQLM[147]SQPSTASNS[167]LPEPAK	1	7	4
	x					681	703	S	697	3	LSQPGQLM[147]SQPSTASNS[167]LPEPAK	1	7	3
		x				681	703	S	697	2	LSQPGQLM[147]SQPSTASNS[167]LPEPAK	1	7	4
		x				681	703	S	697	3	LSQPGQLM[147]SQPSTASNS[167]LPEPAK	1	7	6
				x		681	703	S	697	2	LSQPGQLM[147]SQPSTASNS[167]LPEPAK	0.9999	7	3
				x		681	703	S	697	3	LSQPGQLM[147]SQPSTASNS[167]LPEPAK	0.9997	7	1
x						681	703	S	697	3	LSQPGQLMSQPSTASNS[167]LPEPAK	1	7	3
		x				681	703	S	697	2	LSQPGQLMSQPSTASNS[167]LPEPAK	1	7	4
		x				681	703	S	697	3	LSQPGQLMSQPSTASNS[167]LPEPAK	1	7	4
x						681	703	S	697	2	LSQPGQLMSQPSTASNS[167]LPEPAK	0.9999	7	1
	x					681	703	S	697	2	LSQPGQLMSQPSTASNS[167]LPEPAK	0.9999	7	5
	x					681	703	S	697	3	LSQPGQLMSQPSTASNS[167]LPEPAK	0.9999	7	3
				x		681	703	S	697	2	LSQPGQLMSQPSTASNS[167]LPEPAK	0.9999	7	5
				x		681	703	S	697	3	LSQPGQLMSQPSTASNS[167]LPEPAK	0.9997	7	2
x						681	704	S	697	3	LSQPGQLM[147]SQPSTASNS[167]LPEPAKK	0.9998	7	6
	x					681	704	S	697	3	LSQPGQLM[147]SQPSTASNS[167]LPEPAKK	0.9998	7	7
		x				681	704	S	697	3	LSQPGQLM[147]SQPSTASNS[167]LPEPAKK	0.9998	7	8
				x		681	704	S	697	3	LSQPGQLM[147]SQPSTASNS[167]LPEPAKK	0.9993	7	5
	x					681	704	S	697	2	LSQPGQLM[147]SQPSTASNS[167]LPEPAKK	0.9925	7	1
		x				681	704	S	697	3	LSQPGQLMSQPSTASNS[167]LPEPAKK	0.9999	7	8
	x					681	704	S	697	3	LSQPGQLMSQPSTASNS[167]LPEPAKK	0.9995	7	4
				x		681	704	S	697	2	LSQPGQLMSQPSTASNS[167]LPEPAKK	0.9992	7	1
x						681	704	S	697	3	LSQPGQLMSQPSTASNS[167]LPEPAKK	0.9984	7	4
				x		681	704	S	697	3	LSQPGQLMSQPSTASNS[167]LPEPAKK	0.3764	7	4
			x			720	734	S	733	2	ENAIQDTVREQDQS[167]F	1	7	5

					x	720	734	S	733	2	ENAIQDTVREQDQS[167]F	0.9999	7	5
					x	720	734	S	733	3	ENAIQDTVREQDQS[167]F_ETD	0.9999	7	3
			x			721	734	S	733	2	NAIQDTVREQDQS[167]F	1	7	8
					x	721	734	S	733	2	NAIQDTVREQDQS[167]F	0.9999	7	7
					x	721	734	S	733	3	NAIQDTVREQDQS[167]F_ETD	0.9999	7	2
			x			721	734	S	733	3	NAIQDTVREQDQS[167]F_ETD	0.0035	7	1
			x			721	736	S	733	2	NAIQDTVREQDQS[167]FTA	1	7	6
					x	721	736	S	733	2	NAIQDTVREQDQS[167]FTA	0.9999	7	3
					x	721	736	S	733	3	NAIQDTVREQDQS[167]FTA_ETD	0.9905	7	2
					x	721	737	S	733	2	NAIQDTVREQDQS[167]FTAL	0.9999	7	4
			x			721	737	S	733	2	NAIQDTVREQDQS[167]FTAL	0.998	7	4
					x	721	737	S	733	3	NAIQDTVREQDQS[167]FTAL	0.2221	7	2
			x			722	734	S	733	2	AIQDTVREQDQS[167]F	1	7	6
					x	722	734	S	733	2	AIQDTVREQDQS[167]F	0.9982	7	3
			x			722	737	S	733	2	AIQDTVREQDQS[167]FTAL	1	7	4
					x	722	737	S	733	2	AIQDTVREQDQS[167]FTAL	0.9999	7	4
					x	723	734	S	733	2	IQDTVREQDQS[167]F	0.9999	7	5
			x			723	734	S	733	2	IQDTVREQDQS[167]F	0.9996	7	9
					x	723	734	S	733	3	IQDTVREQDQS[167]F_ETD	0.9999	7	2
			x			723	737	S	733	2	IQDTVREQDQS[167]FTAL	1	7	4
					x	723	737	S	733	2	IQDTVREQDQS[167]FTAL	0.9999	7	4
					x	723	737	S	733	3	IQDTVREQDQS[167]FTAL_ETD	0.9999	7	2
			x			725	737	S	733	2	DTVREQDQS[167]FTAL	0.999	7	4
					x	725	737	S	733	2	DTVREQDQS[167]FTAL	0.9927	7	7
					x	725	737	S	733	3	DTVREQDQS[167]FTAL_ETD	0.9999	7	1
					x	726	737	S	733	2	TVREQDQS[167]FTAL	0.9986	7	19
			x			726	737	S	733	2	TVREQDQS[167]FTAL	0.9946	7	9
					x	726	737	S	733	3	TVREQDQS[167]FTAL_ETD	0.9999	7	2
					x	727	737	S	733	2	VREQDQS[167]FTAL	0.9958	7	3
			x			727	737	S	733	2	VREQDQS[167]FTAL	0.9876	7	2
				x		729	738	S	733	2	EQDQS[167]FTALD	0.0175	7	1
x						729	739	S	733	2	EQDQS[167]FTALDW	0.0086	7	1
x						729	740	S	733	2	EQDQS[167]FTALDWS	0.1205	7	1
	x					729	756	S	733	3	EQDQS[167]FTALDWSWLQTEEEEHSCLEQAS	0.9999	7	1
			x			721	736	T	735	2	NAIQDTVREQDQSFT[181]A	0.9953	7	1
x						729	740	T	735	2	EQDQSFT[181]ALDWS	0.9964	7	5
				x		729	740	T	735	2	EQDQSFT[181]ALDWS	0.3951	7	4
	x					729	740	T	735	2	EQDQSFT[181]ALDWS	0.3089	7	2
		x				729	740	T	735	2	EQDQSFT[181]ALDWS	0.0624	7	1
	x					729	756	T	735	3	EQDQSFT[181]ALDWSWLQTEEEEHSCLEQAS	0.9997	7	1
	x					729	756	S	740	3	EQDQSFTALDWS[167]WLQTEEEEHSCLEQAS	0.0547	7	1
					x	735	744	S	740	2	TALDWS[167]WLQT	0.0143	7	1
				x		580	591	DOUBLE	583+587	2	DQRT[181]EGDS[167]QEM[147]V	0.0006	7	1
				x		665	680	DOUBLE	672+675	2	VRGPVSGS[167]PDS[167]MNASR	0.0112	7	1
				x		681	704	DOUBLE	693+697	3	LSQPGQLM[147]SQPST[181]ASNS[167]LPEPAKK	0.1228	7	1
x						5	18			2	PSLTTQTCGAWEMK	0.0961	7	1
x						21	31			2	LGTGGFGNVIR	1	7	52
	x					21	31			2	LGTGGFGNVIR	1	7	21
		x				21	31			2	LGTGGFGNVIR	1	7	23
				x		21	31			2	LGTGGFGNVIR	0.9999	7	109
					x	27	40			2	GNVIRWHNQETGEQ	0.784	7	1
x						32	44			2	WHNQETGEQIAIK	1	7	13
		x				32	44			2	WHNQETGEQIAIK	1	7	3
				x		32	44			2	WHNQETGEQIAIK	0.9999	7	60
x						32	44			3	WHNQETGEQIAIK_ETD	1	7	10
		x				32	44			3	WHNQETGEQIAIK_ETD	1	7	2
				x		32	44			3	WHNQETGEQIAIK_ETD	0.9999	7	16
				x		32	47			3	WHNQETGEQIAIKQCR	0.438	7	1
		x				58	66			2	WCLEIQIM[147]R	0.9969	7	1
x						58	66			2	WCLEIQIM[147]R	0.9907	7	1
				x		58	66			2	WCLEIQIMR	0.9999	7	6
x						58	66			2	WCLEIQIMR	0.9995	7	2
x						67	77			2	RLTHPNVVAAR	1	7	4
				x		67	77			2	RLTHPNVVAAR	0.9999	7	46
x						67	77			3	RLTHPNVVAAR_ETD	1	7	2
				x		67	77			3	RLTHPNVVAAR_ETD	0.9999	7	49
				x		67	77			4	RLTHPNVVAAR_ETD	0.9992	7	1
x						68	77			2	LTHPNVVAAR	1	7	24

		x				68	77	2	LTHPNVVAAR	1	7	3
				x		68	77	2	LTHPNVVAAR	0.9999	7	34
x						68	77	3	LTHPNVVAAR_ETD	1	7	15
		x				68	77	3	LTHPNVVAAR_ETD	1	7	4
				x		68	77	3	LTHPNVVAAR_ETD	0.9999	7	57
					x	76	91	2	ARDVPEGM[147]QNLAPNDL	0.9862	7	3
			x			76	91	2	ARDVPEGM[147]QNLAPNDL	0.0077	7	1
					x	76	91	2	ARDVPEGMQNLAPNDL	0.3119	7	1
x						78	105	3	DVPEGM[147]QNLAPNDLPLLAM[147]EYCQGGDLR	1	7	8
	x					78	105	3	DVPEGM[147]QNLAPNDLPLLAM[147]EYCQGGDLR	1	7	4
		x				78	105	3	DVPEGM[147]QNLAPNDLPLLAM[147]EYCQGGDLR	1	7	6
				x		78	105	3	DVPEGM[147]QNLAPNDLPLLAM[147]EYCQGGDLR	0.9999	7	3
x						78	105	3	DVPEGM[147]QNLAPNDLPLLAMEYCQGGDLR	1	7	2
				x		78	105	3	DVPEGM[147]QNLAPNDLPLLAMEYCQGGDLR	0.9999	7	6
		x				78	105	3	DVPEGM[147]QNLAPNDLPLLAMEYCQGGDLR	0.9996	7	1
	x					78	105	3	DVPEGM[147]QNLAPNDLPLLAMEYCQGGDLR	0.9992	7	2
	x					78	105	3	DVPEGMQNLAPNDLPLLAM[147]EYCQGGDLR	0.9999	7	1
				x		78	105	3	DVPEGMQNLAPNDLPLLAM[147]EYCQGGDLR	0.9947	7	4
x						78	105	3	DVPEGMQNLAPNDLPLLAM[147]EYCQGGDLR	0.9929	7	1
				x		78	105	3	DVPEGMQNLAPNDLPLLAMEYCQGGDLR	0.9999	7	8
x						78	105	3	DVPEGMQNLAPNDLPLLAMEYCQGGDLR	0.3055	7	1
				x		78	105	4	DVPEGMQNLAPNDLPLLAMEYCQGGDLR_ETD	0.0011	7	1
				x		78	106	3	DVPEGM[147]QNLAPNDLPLLAM[147]EYCQGGDLRK	0.9978	7	4
				x		78	106	3	DVPEGM[147]QNLAPNDLPLLAMEYCQGGDLRK	0.9996	7	4
				x		78	106	3	DVPEGMQNLAPNDLPLLAM[147]EYCQGGDLRK	0.9991	7	5
				x		78	106	3	DVPEGMQNLAPNDLPLLAMEYCQGGDLRK	0.9998	7	7
					x	85	93	2	NLAPNDLPL	0.0493	7	1
				x		106	118	2	KYLNQFENCCGLR	0.9999	7	16
				x		106	118	3	KYLNQFENCCGLR_ETD	0.9999	7	12
x						107	118	2	YLNQFENCCGLR	1	7	14
		x				107	118	2	YLNQFENCCGLR	1	7	6
	x					107	118	2	YLNQFENCCGLR	0.9999	7	2
				x		107	118	2	YLNQFENCCGLR	0.9999	7	25
x						119	134	2	EGAILTLLSDIASALR	1	7	2
		x				119	134	2	EGAILTLLSDIASALR	1	7	3
	x					119	134	2	EGAILTLLSDIASALR	0.9998	7	1
x						119	134	3	EGAILTLLSDIASALR_ETD	1	7	1
					x	124	132	2	TLLSDIASA	0.0091	7	1
					x	124	133	2	TLLSDIASAL	0.276	7	2
					x	126	133	2	LSDIASAL	0.0327	7	2
			x			126	133	2	LSDIASAL	0.0081	7	1
					x	139	150	2	NRIIHRDLKPEN	0.9974	7	29
			x			139	150	2	NRIIHRDLKPEN	0.9918	7	6
			x			139	150	3	NRIIHRDLKPEN_ETD	1	7	3
					x	139	150	3	NRIIHRDLKPEN_ETD	0.3496	7	1
					x	139	150	4	NRIIHRDLKPEN_ETD	0.0068	7	1
				x		145	156	2	DLKPENIVLQQG	0.0221	7	1
x						145	159	2	DLKPENIVLQQGEQR	1	7	10
						145	159	2	DLKPENIVLQQGEQR	1	7	2
		x				145	159	2	DLKPENIVLQQGEQR	1	7	7
				x		145	159	2	DLKPENIVLQQGEQR	0.9999	7	24
x						145	159	3	DLKPENIVLQQGEQR_ETD	1	7	69
		x				145	159	3	DLKPENIVLQQGEQR_ETD	1	7	45
	x					145	159	3	DLKPENIVLQQGEQR_ETD	0.9999	7	52
				x		145	159	3	DLKPENIVLQQGEQR_ETD	0.9999	7	55
			x			151	160	2	IVLQQGEQRL	0.9993	7	9
					x	151	160	2	IVLQQGEQRL	0.9987	7	4
					x	161	168	2	IHKIIDLG	0.356	7	8
			x			161	168	2	IHKIIDLG	0.0027	7	1
					x	161	168	3	IHKIIDLG_ETD	0.9928	7	1
				x		164	171	2	IIDLGYAK	0.9996	7	28
		x				164	171	2	IIDLGYAK	0.9993	7	3
x						164	171	2	IIDLGYAK	0.999	7	19
					x	169	178	2	YAKELDQGSL	0.9999	7	5
			x			169	178	2	YAKELDQGSL	0.8822	7	3
		x				172	182	2	ELDQGSLCTSF	0.069	7	1
				x		172	182	2	ELDQGSLCTSF	0.0067	7	1
		x				172	188	2	ELDQGSLCTSFVGTLQY	0.9979	7	3

x						172	198	3	ELDQGSLCTSFVGTLQYLAPELLEQQK	1	7	1
				x		172	198	3	ELDQGSLCTSFVGTLQYLAPELLEQQK	0.9999	7	2
				x		189	198	2	LAPELLEQQK	0.9929	7	6
			x			194	202	2	LEQQKYTVT	0.9964	7	57
					x	194	202	2	LEQQKYTVT	0.9874	7	5
					x	194	205	2	LEQQKYTVTVDY	0.9999	7	9
					x	220	231	2	RPFLPNWQPVQW	0.9966	7	3
				x		221	234	3	PFLPNWQPVQWHSK_ETD	0.9984	7	1
					x	224	231	2	PNWQPVQW	0.9673	7	8
			x			224	231	2	PNWQPVQW	0.041	7	4
		x				237	254	2	QKSEVDIVVSEDLNGTVK	0.8812	7	1
x						237	254	2	QKSEVDIVVSEDLNGTVK	0.848	7	1
x						239	254	2	SEVDIVVSEDLNGTVK	1	7	17
	x					239	254	2	SEVDIVVSEDLNGTVK	1	7	6
		x				239	254	2	SEVDIVVSEDLNGTVK	1	7	3
				x		239	254	2	SEVDIVVSEDLNGTVK	0.9999	7	12
x						239	254	3	SEVDIVVSEDLNGTVK_ETD	1	7	5
				x		239	254	3	SEVDIVVSEDLNGTVK_ETD	0.9999	7	8
					x	242	255	2	DIVVSEDLNGTVKF	0.9999	7	3
			x			242	255	2	DIVVSEDLNGTVKF	0.9974	7	1
			x			243	255	2	IVVSEDLNGTVKF	1	7	6
					x	243	255	2	IVVSEDLNGTVKF	0.9999	7	11
					x	243	255	3	IVVSEDLNGTVKF_ETD	0.9999	7	2
					x	245	255	2	VSEDLNGTVKF	0.9999	7	3
					x	248	255	2	DLNGTVKF	0.9938	7	5
x						255	263	2	FSSSLPYPN	0.1565	7	2
				x		255	263	2	FSSSLPYPN	0.111	7	2
		x				255	263	2	FSSSLPYPN	0.0108	7	1
		x				255	266	2	FSSSLPYPNNLN	0.7902	7	8
	x					255	266	2	FSSSLPYPNNLN	0.0128	7	3
x						255	272	2	FSSSLPYPNNLNSVLAER	1	7	12
x						255	272	3	FSSSLPYPNNLNSVLAER	1	7	7
		x				255	272	2	FSSSLPYPNNLNSVLAER	1	7	10
				x		255	272	2	FSSSLPYPNNLNSVLAER	0.9999	7	9
				x		255	272	3	FSSSLPYPNNLNSVLAER	0.9999	7	10
	x					255	272	2	FSSSLPYPNNLNSVLAER	0.9991	7	1
	x					255	272	3	FSSSLPYPNNLNSVLAER	0.9712	7	1
				x		255	275	3	FSSSLPYPNNLNSVLAERLEK	0.0184	7	2
			x			256	264	2	SSSLPYPNN	0.0148	7	1
			x			256	265	2	SSSLPYPNNL	0.9972	7	9
					x	256	265	2	SSSLPYPNNL	0.9955	7	7
				x		264	272	2	NLNSVLAER	0.9893	7	9
				x		276	286	2	WLQLMLMWHPR	0.9999	7	1
				x		280	286	2	M[147]LM[147]WHPR	0.0271	7	1
				x		280	286	2	MLM[147]WHPR	0.046	7	1
				x		280	286	3	MLM[147]WHPR_ETD	0.3623	7	3
x						289	301	2	GTDPTYGPNGCFK	1	7	43
		x				289	301	2	GTDPTYGPNGCFK	1	7	37
				x		289	301	2	GTDPTYGPNGCFK	0.9999	7	42
	x					289	301	2	GTDPTYGPNGCFK	0.9998	7	3
				x		290	301	2	TDPTYGPNGCFK	0.0023	7	1
				x		295	301	2	PTYGPNGCFK	0.9922	7	27
					x	301	307	2	KALDDIL	0.9229	7	7
			x			301	307	2	KALDDIL	0.2403	7	5
					x	301	308	2	KALDDILN	0.9967	7	3
			x			301	308	2	KALDDILN	0.9639	7	13
					x	301	309	2	KALDDILNL	0.4468	7	3
x						302	310	2	ALDDILNLK	1	7	38
		x				302	310	2	ALDDILNLK	1	7	38
	x					302	310	2	ALDDILNLK	0.9999	7	12
				x		302	310	2	ALDDILNLK	0.9999	7	54
				x		311	320	2	LVHILNM[147]VTG	0.0096	7	1
x						311	337	3	LVHILNM[147]VTGTIHTYPVTEDESLQSLK	1	7	2
		x				311	337	3	LVHILNM[147]VTGTIHTYPVTEDESLQSLK	1	7	1
				x		311	337	3	LVHILNM[147]VTGTIHTYPVTEDESLQSLK	0.9999	7	5
x						311	337	4	LVHILNM[147]VTGTIHTYPVTEDESLQSLK_ETD	1	7	2
				x		311	337	4	LVHILNM[147]VTGTIHTYPVTEDESLQSLK_ETD	0.9999	7	8
x						311	337	3	LVHILNMVTGTIHTYPVTEDESLQSLK	1	7	1

		x				311	337	3	LVHILNMVTGTIHTYPVTEDESLQSLK	1	7	1
				x		311	337	3	LVHILNMVTGTIHTYPVTEDESLQSLK	0.9999	7	6
x						311	337	4	LVHILNMVTGTIHTYPVTEDESLQSLK_ETD	0.9999	7	1
				x		311	337	4	LVHILNMVTGTIHTYPVTEDESLQSLK_ETD	0.9999	7	8
					x	316	330	2	NM[147]VTGTIHTYPVTED	0.9939	7	2
					x	317	229	2	M[147]VTGTIHTYPVTE	0.9999	7	9
					x	317	229	2	MVTGTIHTYPVTE	0.9999	7	2
					x	317	331	2	M[147]VTGTIHTYPVTEDE	0.9969	7	3
					x	317	333	2	M[147]VTGTIHTYPVTEDESL	0.9999	7	1
					x	317	333	2	MVTGTIHTYPVTEDESL	0.8242	7	1
x						317	337	3	M[147]VTGTIHTYPVTEDESLQSLK	0.0229	7	1
x						317	337	3	MVTGTIHTYPVTEDESLQSLK	1	7	5
		x				317	337	3	MVTGTIHTYPVTEDESLQSLK	0.9992	7	1
					x	318	329	2	VTGTIHTYPVTE	0.9973	7	13
			x			318	329	2	VTGTIHTYPVTE	0.8335	7	20
					x	318	331	2	VTGTIHTYPVTEDE	0.9999	7	4
					x	318	333	2	VTGTIHTYPVTEDESL	0.9999	7	4
			x			318	333	2	VTGTIHTYPVTEDESL	0.9985	7	7
				x		326	337	2	PVTEDESLQSLK	0.9771	7	3
		x				326	337	2	PVTEDESLQSLK	0.2738	7	3
					x	337	349	2	KARIQQDTGIPEE	0.2111	7	1
			x			339	352	2	RIQQDTGIPEEDQE	0.1911	7	3
x						340	375	3	IQQDTGIPEEDQELLQEAGLALIPDKPATQCISDGK	0.9999	7	3
		x				340	375	3	IQQDTGIPEEDQELLQEAGLALIPDKPATQCISDGK	0.9999	7	3
				x		340	375	3	IQQDTGIPEEDQELLQEAGLALIPDKPATQCISDGK	0.9999	7	13
x						340	375	4	IQQDTGIPEEDQELLQEAGLALIPDKPATQCISDGK	0.9995	7	1
	x					340	375	4	IQQDTGIPEEDQELLQEAGLALIPDKPATQCISDGK	0.9971	7	1
	x					340	375	3	IQQDTGIPEEDQELLQEAGLALIPDKPATQCISDGK	0.9969	7	2
				x		340	375	4	IQQDTGIPEEDQELLQEAGLALIPDKPATQCISDGK	0.9861	7	5
					x	360	369	2	ALIPDKPATQ	0.9796	7	8
			x			360	369	2	ALIPDKPATQ	0.145	7	11
					x	360	374	2	ALIPDKPATQCISDG	0.0275	7	1
x						376	394	2	LNEGHTLDM[147]DLVFLFDNSK	1	7	2
		x				376	394	2	LNEGHTLDM[147]DLVFLFDNSK	1	7	2
		x				376	394	3	LNEGHTLDM[147]DLVFLFDNSK	1	7	5
x						376	394	3	LNEGHTLDM[147]DLVFLFDNSK	0.9998	7	2
				x		376	394	3	LNEGHTLDM[147]DLVFLFDNSK	0.9998	7	4
		x				376	394	2	LNEGHTLDMDLVFLFDNSK	1	7	3
		x				376	394	3	LNEGHTLDMDLVFLFDNSK	0.9999	7	2
				x		376	394	2	LNEGHTLDMDLVFLFDNSK	0.9999	7	5
				x		376	394	3	LNEGHTLDMDLVFLFDNSK	0.9979	7	6
	x					376	394	3	LNEGHTLDMDLVFLFDNSK	0.9952	7	1
					x	377	386	2	NEGHTLDM[147]DL	0.2105	7	1
					x	377	386	2	NEGHTLDMDL	0.9813	7	6
					x	389	397	2	LFDNSKITY	0.9999	7	8
					x	389	398	2	LFDNSKITYE	0.9999	7	7
			x			389	398	2	LFDNSKITYE	0.9995	7	3
					x	390	397	2	FDNSKITY	0.8903	7	1
			x			390	397	2	FDNSKITY	0.0012	7	1
					x	390	398	2	FDNSKITYE	0.9999	7	8
			x			390	398	2	FDNSKITYE	0.9991	7	59
					x	390	400	2	FDNSKITYETQ	0.9984	7	2
			x			390	400	2	FDNSKITYETQ	0.9094	7	2
x						395	404	2	ITYETQISPR	1	7	50
	x					395	404	2	ITYETQISPR	1	7	4
		x				395	404	2	ITYETQISPR	1	7	40
				x		395	404	2	ITYETQISPR	0.9999	7	35
		x				395	412	2	ITYETQISPRPQPESVSC	1	7	4
				x		395	412	2	ITYETQISPRPQPESVSC	0.9999	7	8
				x		395	412	3	ITYETQISPRPQPESVSC	0.7757	7	2
x						395	418	3	ITYETQISPRPQPESVSCILQEPK	0.9999	7	6
				x		395	418	3	ITYETQISPRPQPESVSCILQEPK	0.9999	7	24
				x		395	418	2	ITYETQISPRPQPESVSCILQEPK	0.9977	7	1
		x				395	418	3	ITYETQISPRPQPESVSCILQEPK	0.9966	7	2
				x		395	418	4	ITYETQISPRPQPESVSCILQEPK_ETD	0.9999	7	11
				x		398	418	3	ETQISPRPQPESVSCILQEPK	0.0752	7	1
			x			399	409	2	TQISPRPQPES	0.7762	7	3
					x	399	409	2	TQISPRPQPES	0.0222	7	2

					x	399	410	2	TQISPRPQPESV	0.9999	7	16
			x			399	410	2	TQISPRPQPESV	0.9927	7	58
					x	399	411	2	TQISPRPQPESVS	0.1749	7	8
x						405	418	2	PQPESVSCILQEPK	1	7	26
	x					405	418	2	PQPESVSCILQEPK	1	7	3
		x				405	418	2	PQPESVSCILQEPK	1	7	30
				x		405	418	2	PQPESVSCILQEPK	0.9999	7	14
		x				405	418	3	PQPESVSCILQEPK_ETD	0.9985	7	3
				x		405	418	3	PQPESVSCILQEPK_ETD	0.9982	7	9
				x		419	426	2	RNLAFFQLR	0.9999	7	19
x						419	426	2	RNLAFFQLR	0.9997	7	1
				x		419	426	3	RNLAFFQLR_ETD	0.9999	7	6
x						420	426	2	NLAFFQLR	1	7	7
				x		420	426	2	NLAFFQLR	0.9999	7	25
		x				420	426	2	NLAFFQLR	0.9998	7	4
	x					420	426	2	NLAFFQLR	0.9853	7	1
				x		428	435	2	KVWGQVWH	0.7797	7	7
				x		428	435	3	KVWGQVWH_ETD	0.999	7	1
				x		428	441	3	KVWGQVWHSIQTLK_ETD	0.9989	7	1
				x		429	435	2	VWGQVWH	0.0924	7	2
x						429	441	2	VWGQVWHSIQTLK	0.9995	7	1
					x	432	440	2	QVWHSIQTL	0.026	7	1
x						453	460	2	AAM[147]M[147]NLLR	0.9999	7	9
				x		453	460	2	AAM[147]M[147]NLLR	0.9999	7	17
	x					453	460	2	AAM[147]M[147]NLLR	0.9985	7	3
		x				453	460	2	AAM[147]M[147]NLLR	0.9582	7	1
x						453	460	2	AAMM[147]NLLR	1	7	11
		x				453	460	2	AAMM[147]NLLR	1	7	4
				x		453	460	2	AAMM[147]NLLR	0.9999	7	33
				x		453	460	2	AAMMNLLR	0.9999	7	22
x						453	460	2	AAMMNLLR	0.9961	7	1
				x		468	480	2	MKNSM[147]ASM[147]SQQLK	0.0211	7	1
				x		468	480	2	MKNSM[147]ASMSQQLK	0.9958	7	4
				x		468	480	2	MKNSMASM[147]SQQLK	0.998	7	3
				x		468	480	2	MKNSMASMSQQLK	0.9999	7	9
x						470	480	2	NSM[147]ASM[147]SQQLK	0.9997	7	1
x						470	480	2	NSM[147]ASMSQQLK	1	7	15
		x				470	480	2	NSM[147]ASMSQQLK	1	7	11
				x		470	480	2	NSM[147]ASMSQQLK	0.9999	7	24
x						470	480	2	NSMASM[147]SQQLK	1	7	5
		x				470	480	2	NSMASM[147]SQQLK	1	7	16
				x		470	480	2	NSMASM[147]SQQLK	0.9999	7	13
	x					470	480	2	NSMASM[147]SQQLK	0.9998	7	1
x						470	480	2	NSMASMSQQLK	1	7	11
		x				470	480	2	NSMASMSQQLK	1	7	24
				x		470	480	2	NSMASMSQQLK	0.9999	7	31
				x		470	480	3	NSMASMSQQLK_ETD	0.9939	7	1
					x	476	485	2	SQQLKAKLDF	0.4277	7	1
				x		481	487	2	AKLDFFK	0.9962	7	22
x						481	487	2	AKLDFFK	0.0199	7	1
					x	486	493	2	FKTSIQID	0.3346	7	1
x						488	496	2	TSIQIDLEK	0.9999	7	39
		x				488	496	2	TSIQIDLEK	0.9998	7	14
	x					488	496	2	TSIQIDLEK	0.9996	7	5
				x		488	496	2	TSIQIDLEK	0.9996	7	21
				x		488	509	2	TSIQIDLEKYSEQTEFGITSDK	0.9999	7	8
				x		488	509	3	TSIQIDLEKYSEQTEFGITSDK	0.9998	7	12
x						497	509	2	YSEQTEFGITSDK	1	7	58
	x					497	509	2	YSEQTEFGITSDK	1	7	13
		x				497	509	2	YSEQTEFGITSDK	1	7	62
				x		497	509	2	YSEQTEFGITSDK	0.9999	7	50
					x	501	510	2	TEFGITSDKL	0.2014	7	4
			x			501	510	2	TEFGITSDKL	0.0006	7	1
					x	503	510	2	FGITSDKL	0.9986	7	2
					x	504	511	2	GITSDKLL	0.0811	7	1
					x	504	512	2	GITSDKLLL	0.9948	7	8
			x			504	512	2	GITSDKLLL	0.0176	7	1
					x	513	522	2	AWREM[147]EQAVE	0.981	7	1

					x	514	522	2	WREMEQAVE	0.0184	7	1
x						516	526	2	EM[147]EQAVELCGR	1	7	48
	x					516	526	2	EM[147]EQAVELCGR	1	7	4
		x				516	526	2	EM[147]EQAVELCGR	1	7	49
				x		516	526	2	EM[147]EQAVELCGR	0.9999	7	44
x						516	526	2	EMEQAVELCGR	1	7	45
	x					516	526	2	EMEQAVELCGR	1	7	6
		x				516	526	2	EMEQAVELCGR	1	7	40
				x		516	526	2	EMEQAVELCGR	0.9999	7	31
				x		516	526	3	EMEQAVELCGR_ETD	0.9999	7	2
				x		516	531	2	EMEQAVELCGRENEVK	0.9995	7	10
				x		516	531	3	EMEQAVELCGRENEVK_ETD	0.2537	7	1
x						537	549	2	M[147]M[147]ALQTDIVDLQR	1	7	19
	x					537	549	2	M[147]M[147]ALQTDIVDLQR	1	7	7
		x				537	549	2	M[147]M[147]ALQTDIVDLQR	1	7	23
				x		537	549	2	M[147]M[147]ALQTDIVDLQR	0.9999	7	14
x						537	549	2	M[147]MALQTDIVDLQR	1	7	15
	x					537	549	2	M[147]MALQTDIVDLQR	1	7	5
		x				537	549	2	M[147]MALQTDIVDLQR	1	7	6
				x		537	549	2	M[147]MALQTDIVDLQR	0.9999	7	25
x						537	549	2	MM[147]ALQTDIVDLQR	1	7	11
	x					537	549	2	MM[147]ALQTDIVDLQR	1	7	1
		x				537	549	2	MM[147]ALQTDIVDLQR	1	7	5
				x		537	549	2	MM[147]ALQTDIVDLQR	0.9999	7	13
				x		537	549	3	MM[147]ALQTDIVDLQR_ETD	0.9999	7	10
x						537	549	2	MMALQTDIVDLQR	1	7	9
		x				537	549	2	MMALQTDIVDLQR	1	7	3
				x		537	549	2	MMALQTDIVDLQR	0.9999	7	19
				x		537	549	3	MMALQTDIVDLQR_ETD	0.9999	7	8
				x		539	549	2	ALQTDIVDLQR	0.9964	7	6
x						541	549	2	QTDIVDLQR	0.9903	7	6
		x				541	549	2	QTDIVDLQR	0.9901	7	18
				x		541	549	2	QTDIVDLQR	0.92	7	14
					x	548	560	3	QRSPM[147]GRKQGGTL_ETD	0.9884	7	1
x						555	568	2	KQGGTLDDLEEQAR	1	7	20
		x				555	568	2	KQGGTLDDLEEQAR	1	7	6
				x		555	568	2	KQGGTLDDLEEQAR	0.9999	7	71
x						555	568	3	KQGGTLDDLEEQAR_ETD	1	7	9
		x				555	568	3	KQGGTLDDLEEQAR_ETD	1	7	2
				x		555	568	3	KQGGTLDDLEEQAR_ETD	0.9999	7	43
				x		555	572	3	KQGGTLDDLEEQARELYR	0.9982	7	1
x						556	568	2	QGGTLDDLEEQAR	1	7	48
	x					556	568	2	QGGTLDDLEEQAR	1	7	15
		x				556	568	2	QGGTLDDLEEQAR	1	7	62
				x		556	568	2	QGGTLDDLEEQAR	0.9999	7	23
				x		556	568	3	QGGTLDDLEEQAR_ETD	0.9999	7	1
				x		558	568	2	GTLDDLEEQAR	0.0976	7	1
				x		559	568	2	TLDDLEEQAR	0.2115	7	2
				x		583	592	2	TEGDSQEMVR	0.9999	7	2
				x		593	601	2	LLLQAIQSF	0.9956	7	3
x						593	603	2	LLLQAIQSFEK	1	7	24
		x				593	603	2	LLLQAIQSFEK	1	7	56
	x					593	603	2	LLLQAIQSFEK	0.9999	7	10
				x		593	603	2	LLLQAIQSFEK	0.9999	7	97
				x		593	604	2	LLLQAIQSFEKK	0.9999	7	16
x						593	604	2	LLLQAIQSFEKK	0.9977	7	1
				x		593	604	3	LLLQAIQSFEKK_ETD	0.9999	7	17
			x			602	609	2	EKKVRVIY	0.0562	7	3
					x	602	609	2	EKKVRVIY	0.0162	7	2
			x			602	609	3	EKKVRVIY_ETD	0.9995	7	1
					x	602	609	3	EKKVRVIY_ETD	0.2023	7	1
				x		605	614	2	VRVIYTQLSK	0.8559	7	4
				x		605	614	3	VRVIYTQLSK_ETD	0.9997	7	1
x						607	614	2	VIYTQLSK	0.9998	7	25
		x				607	614	2	VIYTQLSK	0.9995	7	22
				x		607	614	2	VIYTQLSK	0.9995	7	35
	x					607	614	2	VIYTQLSK	0.9984	7	2
x						622	627	2	ALELLPK	0.9999	7	19

		x				622	627	2	ALELLPK	0.9998	7	4
				x		622	627	2	ALELLPK	0.9997	7	32
				x		622	641	3	ALELLPKVEEVVSLM[147]NEDEK	0.9948	7	4
				x		622	641	3	ALELLPKVEEVVSLMNEDEK	0.9959	7	4
				x		622	645	3	ALELLPKVEEVVSLM[147]NEDEKTVVR	0.9999	7	4
				x		622	645	4	ALELLPKVEEVVSLM[147]NEDEKTVVR_ETD	0.9993	7	5
				x		622	645	3	ALELLPKVEEVVSLMNEDEKTVVR	0.9999	7	3
				x		622	645	4	ALELLPKVEEVVSLMNEDEKTVVR_ETD	0.9963	7	2
x						629	641	2	VEEVVSLM[147]NEDEK	1	7	60
	x					629	641	2	VEEVVSLM[147]NEDEK	1	7	12
		x				629	641	2	VEEVVSLM[147]NEDEK	1	7	62
				x		629	641	2	VEEVVSLM[147]NEDEK	0.9999	7	37
x						629	641	3	VEEVVSLM[147]NEDEK_ETD	1	7	7
		x				629	641	3	VEEVVSLM[147]NEDEK_ETD	1	7	3
				x		629	641	3	VEEVVSLM[147]NEDEK_ETD	0.9999	7	6
	x					629	641	3	VEEVVSLM[147]NEDEK_ETD	0.9943	7	1
x						629	641	2	VEEVVSLMNEDEK	1	7	72
	x					629	641	2	VEEVVSLMNEDEK	1	7	29
		x				629	641	2	VEEVVSLMNEDEK	1	7	27
				x		629	641	2	VEEVVSLMNEDEK	0.9999	7	27
x						629	641	3	VEEVVSLMNEDEK_ETD	1	7	2
				x		629	641	3	VEEVVSLMNEDEK_ETD	0.9999	7	9
			x			636	646	2	M[147]NEDEKTVVRL	1	7	11
					x	636	646	2	M[147]NEDEKTVVRL	0.9999	7	8
					x	636	646	2	MNEDEKTVVRL	0.9999	7	6
			x			636	646	2	MNEDEKTVVRL	0.9993	7	20
				x		651	659	2	QKELWNLLK	0.3738	7	20
				x		651	659	3	QKELWNLLK_ETD	0.9999	7	19
x						651	659	3	QKELWNLLK_ETD	0.9997	7	2
				x		653	659	2	ELWNLLK	0.9933	7	16
		x				653	659	2	ELWNLLK	0.9874	7	1
					x	664	687	3	KVRGPVSGSPDSM[147]NASRLSQPGQL	0.0115	7	2
				x		665	680	2	VRGPVSGSPDSM[147]NASR	0.9991	7	43
				x		665	680	3	VRGPVSGSPDSM[147]NASR_ETD	0.9999	7	44
				x		665	680	2	VRGPVSGSPDSMNASR	0.9999	7	20
				x		665	680	3	VRGPVSGSPDSMNASR_ETD	0.9999	7	1
x						667	680	2	GPVSGSPDSM[147]NASR	1	7	2
		x				667	680	2	GPVSGSPDSM[147]NASR	0.9999	7	9
				x		667	680	2	GPVSGSPDSM[147]NASR	0.9967	7	1
x						667	680	2	GPVSGSPDSMNASR	1	7	2
		x				667	680	2	GPVSGSPDSMNASR	1	7	4
				x		667	680	2	GPVSGSPDSMNASR	0.9999	7	32
			x			668	687	2	PVSGSPDSM[147]NASRLSQPGQL	0.3862	7	3
					x	668	687	2	PVSGSPDSM[147]NASRLSQPGQL	0.3136	7	1
					x	676	687	2	M[147]NASRLSQPGQL	0.0493	7	1
					x	677	687	2	NASRLSQPGQL	0.9783	7	7
					x	678	687	2	ASRLSQPGQL	0.9759	7	5
			x			678	687	2	ASRLSQPGQL	0.0158	7	2
					x	679	687	2	SRLSQPGQL	0.9104	7	4
				x		681	690	2	LSQPGQLM[147]SQ	0.0565	7	1
				x		681	693	2	LSQPGQLM[147]SQPST	0.2379	7	4
				x		681	695	2	LSQPGQLM[147]SQPSTAS	0.4224	7	4
				x		681	695	2	LSQPGQLMSQPSTAS	0.1286	7	1
x						681	696	2	LSQPGQLM[147]SQPSTASN	1	7	39
	x					681	696	2	LSQPGQLM[147]SQPSTASN	1	7	58
		x				681	696	2	LSQPGQLM[147]SQPSTASN	1	7	42
				x		681	696	2	LSQPGQLM[147]SQPSTASN	0.9999	7	13
x						681	696	2	LSQPGQLMSQPSTASN	1	7	14
	x					681	696	2	LSQPGQLMSQPSTASN	1	7	20
		x				681	696	2	LSQPGQLMSQPSTASN	1	7	10
				x		681	696	2	LSQPGQLMSQPSTASN	0.9961	7	6
x						681	703	2	LSQPGQLM[147]SQPSTASNSLPEPAK	1	7	7
		x				681	703	2	LSQPGQLM[147]SQPSTASNSLPEPAK	1	7	33
		x				681	703	3	LSQPGQLM[147]SQPSTASNSLPEPAK	1	7	24
x						681	703	3	LSQPGQLM[147]SQPSTASNSLPEPAK	0.9999	7	1
				x		681	703	2	LSQPGQLM[147]SQPSTASNSLPEPAK	0.9999	7	9
				x		681	703	3	LSQPGQLM[147]SQPSTASNSLPEPAK	0.9996	7	2
		x				681	703	2	LSQPGQLMSQPSTASNSLPEPAK	1	7	24

		x				681	703	3	LSQPGQLMSQPSTASNSLPEPAK	1	7	7
x						681	703	2	LSQPGQLMSQPSTASNSLPEPAK	0.9999	7	1
				x		681	703	2	LSQPGQLMSQPSTASNSLPEPAK	0.9999	7	7
				x		681	703	3	LSQPGQLMSQPSTASNSLPEPAK	0.9999	7	6
		x				681	704	2	LSQPGQLM[147]SQPSTASNSLPEPAKK	0.9999	7	1
				x		681	704	2	LSQPGQLM[147]SQPSTASNSLPEPAKK	0.9999	7	4
x						681	704	3	LSQPGQLM[147]SQPSTASNSLPEPAKK	0.9998	7	17
				x		681	704	3	LSQPGQLM[147]SQPSTASNSLPEPAKK	0.9997	7	30
		x				681	704	3	LSQPGQLM[147]SQPSTASNSLPEPAKK	0.9993	7	19
	x					681	704	3	LSQPGQLM[147]SQPSTASNSLPEPAKK	0.9828	7	2
				x		681	704	2	LSQPGQLMSQPSTASNSLPEPAKK	0.9999	7	5
		x				681	704	2	LSQPGQLMSQPSTASNSLPEPAKK	0.9998	7	1
				x		681	704	3	LSQPGQLMSQPSTASNSLPEPAKK	0.9992	7	18
x						681	704	3	LSQPGQLMSQPSTASNSLPEPAKK	0.9908	7	6
		x				681	704	3	LSQPGQLMSQPSTASNSLPEPAKK	0.9531	7	1
			x			688	699	2	M[147]SQPSTASNSLP	0.9875	7	12
					x	688	703	2	M[147]SQPSTASNSLPEPAK	0.0004	7	1
		x				704	728	3	KSEELVAEAHNLCTLLENAIQDTVR	1	7	1
x						704	728	4	KSEELVAEAHNLCTLLENAIQDTVR_ETD	0.9999	7	1
		x				705	716	2	SEELVAEAHNLC	0.9954	7	5
				x		705	716	2	SEELVAEAHNLC	0.0931	7	2
		x				705	728	3	SEELVAEAHNLCTLLENAIQDTVR	0.9985	7	2
x						705	728	3	SEELVAEAHNLCTLLENAIQDTVR	0.9973	7	1
				x		717	728	2	TLLENAIQDTVR	0.9958	7	1
			x			720	734	2	ENAIQDTVREQDQSF	1	7	16
					x	720	734	2	ENAIQDTVREQDQSF	0.9999	7	10
					x	720	734	3	ENAIQDTVREQDQSF_ETD	0.9985	7	1
			x			721	734	2	NAIQDTVREQDQSF	1	7	19
					x	721	734	2	NAIQDTVREQDQSF	0.9999	7	10
					x	721	734	3	NAIQDTVREQDQSF_ETD	0.9999	7	3
					x	722	734	2	AIQDTVREQDQSF	0.9999	7	4
					x	723	734	2	IQDTVREQDQSF	0.9999	7	4
			x			726	734	2	TVREQDQSF	0.9803	7	11
					x	726	734	2	TVREQDQSF	0.9098	7	3
				x		729	738	2	EQDQSFTALD	0.0573	7	3
x						729	738	2	EQDQSFTALD	0.0077	7	2
				x		729	740	2	EQDQSFTALDWS	0.2663	7	3
x						729	740	2	EQDQSFTALDWS	0.2079	7	1
	x					729	756	3	EQDQSFTALDWSWLQTEEEEHSCLEQAS	1	7	3
		x				729	756	3	EQDQSFTALDWSWLQTEEEEHSCLEQAS	1	7	3
x						729	756	3	EQDQSFTALDWSWLQTEEEEHSCLEQAS	0.9999	7	4
				x		729	756	3	EQDQSFTALDWSWLQTEEEEHSCLEQAS	0.9999	7	6
		x				742	756	2	LQTEEEEHSCLEQAS	0.9964	7	5
x						742	756	2	LQTEEEEHSCLEQAS	0.9958	7	3
				x		742	756	2	LQTEEEEHSCLEQAS	0.9954	7	11
	x					742	756	2	LQTEEEEHSCLEQAS	0.0813	7	2
				x		743	756	2	QTEEEEHSCLEQAS	0.0656	7	1

Notes:

For details of individual preps, see Supplementary Table S1.
Peptides presented with all phospho-peptides first, followed by non-phosphorylated peptides.
Phosphopeptides sorted by: P-amino acid residue; starting residue number; ending residue number; peptide sequence (takes into account M[147] and _ETD); NSP adjusted probability (descending).
Non-phospho peptides sorted by: starting residue number; ending residue number; peptide sequence (takes into account M[147] and _ETD); NSP adjusted probability (descending).

Y[243] indicates pTyr residue in peptide. Shown in Red.
T[181] indicates pThr residue in peptide. Shown in Green.
S[167] indicates pSer residue in peptide. Shown in Blue.
M[147] indicates peptide containing oxidized Met residue.
_ETD indicates peptide identification via an Electron-Transfer Dissociation (ETD) MS/MS spectrum.
