# Supplementary material for: Tyrosine Phosphorylation Allows Integration of Multiple Signaling Inputs by IKKβ
Source: PLoS One. 2013 Dec 27;8(12):e84497. doi: 10.1371/journal.pone.0084497 (PMC3873999; doi:10.1371/journal.pone.0084497)
Supplement: Table S5 — Serine Phosphorylation Sites by Spectral Counts. (PDF) [file pone.0084497.s007.pdf]

Table S5: Serine Phosphorylation Sites by Spectral Counts

Sorted by Total Spectral Count

Residue	Total Spectral Count	%
**S335**	153	14%
**S733**	144	13%
**S672**	140	13%
**S402**	125	12%
**S634**	106	10%
**S697**	100	9%
**S332**	56	5%
**S267**	41	4%
**S695**	24	2%
**S550**	23	2%
**S682**	22	2%
**S246**	21	2%
**S409**	18	2%
**S600**	18	2%
**S258**	16	1%
**S257**	12	1%
**S181**	11	1%
**S670**	8	<1%
**S474**	6	<1%
**S239**	5	<1%
**S256**	5	<1%
**S4**	4	<1%
**S6**	3	<1%
**S675**	3	<1%
**S393**	2	<1%
**S411**	2	<1%
**S476**	2	<1%
**S507**	2	<1%
**S740**	1	<1%

Sorted by Residue Number

Residue	Total Spectral Count	%
**S4**	4	<1%
**S6**	3	<1%
**S181**	11	1%
**S239**	5	<1%
**S246**	21	2%
**S256**	5	<1%
**S257**	12	1%
**S258**	16	1%
**S267**	41	4%
**S332**	56	5%
**S335**	153	14%
**S393**	2	<1%
**S402**	125	12%
**S409**	18	2%
**S411**	2	<1%
**S474**	6	<1%
**S476**	2	<1%
**S507**	2	<1%
**S550**	23	2%
**S600**	18	2%
**S634**	106	10%
**S670**	8	<1%
**S672**	140	13%
**S675**	3	<1%
**S682**	22	2%
**S695**	24	2%
**S697**	100	9%
**S733**	144	13%
**S740**	1	<1%
